# Comparative effects of sulphonylureas, dipeptidyl peptidase‐4 inhibitors and sodium‐glucose co‐transporter‐2 inhibitors added to metformin monotherapy: a propensity‐score matched cohort study in UK primary care

**DOI:** 10.1111/dom.13970

**Published:** 2020-02-13

**Authors:** Samantha Wilkinson, Elizabeth Williamson, Ana Pokrajac, Damian Fogarty, Heide Stirnadel‐Farrant, Liam Smeeth, Ian J. Douglas, Laurie A. Tomlinson

**Affiliations:** ^1^ Department of Non‐Communicable Disease Epidemiology London School of Hygiene and Tropical Medicine London UK; ^2^ West Herts Hospitals NHS Trust Watford UK; ^3^ Belfast Health and Social Care Trust Belfast UK; ^4^ Epidemiology, GlaxoSmithKline Stevenage UK

## Abstract

**Aim:**

To assess the comparative effects of sodium‐glucose co‐transporter‐2 (SGLT2) inhibitors, sulphonylureas (SUs) and dipeptidyl peptidase‐4 (DPP‐4) inhibitors on cardiometabolic risk factors in routine care.

**Materials and methods:**

Using primary care data on 10 631 new users of SUs, SGLT2 inhibitors or DPP‐4 inhibitors added to metformin, obtained from the UK Clinical Practice Research Datalink, we created propensity‐score matched cohorts and used linear mixed models to describe changes in glycated haemoglobin (HbA1c), estimated glomerular filtration rate (eGFR), systolic blood pressure (BP) and body mass index (BMI) over 96 weeks.

**Results:**

HbA1c levels fell substantially after treatment intensification for all drugs: mean change at week 12: SGLT2 inhibitors: −15.2 mmol/mol (95% confidence interval [CI] –16.9, −13.5); SUs: −14.3 mmol/mol (95% CI –15.5, −13.2); and DPP‐4 inhibitors: −11.9 mmol/mol (95% CI –13.1, −10.6). Systolic BP fell for SGLT2 inhibitor users throughout follow‐up, but not for DPP‐4 inhibitor or SU users: mean change at week 12: SGLT2 inhibitors: −2.3 mmHg (95% CI –3.8, −0.8); SUs: −0.8 mmHg (95% CI –1.9, +0.4); and DPP‐4 inhibitors: −0.9 mmHg (95% CI –2.1,+0.2).

BMI decreased for SGLT2 inhibitor and DPP‐4 inhibitor users, but not SU users: mean change at week 12: SGLT2 inhibitors: −0.7 kg/m^2^ (95% CI –0.9, −0.5); SUs: 0.0 kg/m^2^ (95% CI –0.3, +0.2); and DPP‐4 inhibitors: −0.3 kg/m^2^ (95% CI –0.5, −0.1). eGFR fell at 12 weeks for SGLT2 inhibitor and DPP‐4 inhibitor users. At 60 weeks, the fall in eGFR from baseline was similar for each drug class.

**Conclusions:**

In routine care, SGLT2 inhibitors had greater effects on cardiometabolic risk factors than SUs. Routine care data closely replicated the effects of diabetes drugs on physiological variables measured in clinical trials.

## 1 INTRODUCTION

Type 2 diabetes mellitus is a leading cause of morbidity and mortality worldwide, resulting in one million deaths worldwide in 2017.^1^ Drug treatments often provide benefits for glycaemic control and surrogate outcomes but, recently, clinical trials of sodium‐glucose co‐transporter 2 (SGLT2) inhibitors have shown substantial reductions in adverse cardiovascular and renal outcomes.^2^, ^3^, ^4^, ^5^ In these major outcome trials, SGLT2 inhibitors have been compared to placebo, contrasting with the way the drugs have been recommended for use in clinical practice: international guidelines have recommended SGLT2 inhibitors as an option to intensify glycaemic control after metformin monotherapy, but with sulphonylureas (SUs), thiazolidinediones, dipeptidyl peptidase‐4 (DPP‐4) inhibitors or glucagon‐like peptide‐1 (GLP‐1) receptor agonists as alternate choices.^6^, ^7^

The SGLT2 inhibitors work by inhibiting reabsorption of glucose in the proximal renal tubule and thus lowering blood glucose levels. As well as improved glycaemic control, this results in weight loss, blood pressure reduction and diuresis.^8^ In clinical trials of SGLT2 inhibitors, patients in the active treatment arm have shown lower blood pressure and better glycaemic control compared to patients in the placebo arm.^2^, ^3^, ^4^, ^5^ There is limited evidence, however, that lower blood pressure or tighter diabetic control is associated with better cardiovascular outcomes^9^, ^10^; therefore, it is not clear whether the improved clinical outcomes in SGLT2 inhibitor‐treated patients are explained by improvements in known cardiovascular and renal risk factors, which might also occur for other drug classes in direct comparator trials, or whether other mechanisms exist.^11^

Observational studies have compared major outcomes in SGLT2 inhibitor users with those in people who have no additional treatment, and also with those in people using active comparator agents.^12^, ^13^, ^14^, ^15^, ^16^, ^17^ These studies also report substantial outcome benefits for SGLT2 inhibitor users but have been criticised for failing to adequately account for sources of bias and confounding, in particular, for the fact that SGLT2 inhibitors were prescribed to younger patients with fewer comorbidities.^18^ Only few observational studies have examined the effects of first‐line intensification drugs for type 2 diabetes on biological variables and these have mainly focused on the comparative effects of drug classes on glycaemic control.^19^, ^20^, ^21^ The effects of SGLT2 inhibitor drugs on physiological variables, such as blood pressure, measured in routine care, and how these relate to the results observed within the standardized setting of clinical trials, are currently unknown.

The use of DPP‐4 or SGLT2 inhibitors for first‐stage intensification of control of type 2 diabetes has been increasing rapidly in routine clinical care over recent years, with wide variation in prescribing patterns.^22^ There has been relative equipoise for choice of intensification drug offered by current clinical guidelines, and limited differences in the characteristics of people prescribed different drugs which are well understood and measureable.^23^ This combination of circumstances means that observational data lend themselves to a natural experiment: making direct comparisons of medication effects on important diabetes outcomes in a routine care population at the first stage of treatment intensification when SGLT2 inhibitors are commonly used.

Incentivised by the Quality Outcomes Framework, people with type 2 diabetes are regularly monitored in UK primary care, and measures of diabetic control, cardiovascular risk and renal function are recorded well in routine data.^24^ We conducted a propensity‐score matched, new‐user cohort study to determine the effects of the three most commonly used drugs for intensification of glycaemic control after metformin monotherapy, SGLT2 inhibitors, DPP‐4 inhibitors and SUs, on measures of cardiovascular and renal risk.^22^

## 2 MATERIALS AND METHODS

### 2.1 Data sources

We used data from the Clinical Practice Research Datalink (CPRD), which covers ~7% of the UK population and is representative in terms of age, sex and ethnicity.^25^ The data contain information collected by general practitioners and primary care practitioners for routine patient care in primary care settings. Data collected include demographic information, medical diagnoses, prescriptions, laboratory test results and diagnoses made in secondary care. Our data were linked to patient‐level quintiles of index of multiple deprivation (IMD) scores collated in 2015 as a measure of socioeconomic deprivation, provided by the Office of National Statistics.^26^

### 2.2 Study population

To reflect prescribing of drugs used to intensify treatment of type 2 diabetes in contemporary routine clinical practice, we selected a new‐user cohort of adults adding additional treatment to metformin monotherapy (study population). We first identified a study population of individuals aged ≥18 years with a new record of metformin use before any other antidiabetic medication between January 2000 and July 2017. We restricted the study to people with a minimum of 12 months of prior registration in the CPRD to allow complete data entry and to ensure they were new‐users of antidiabetic drugs. From this group, we identified people prescribed one of the potential antidiabetic drug choices recommended by the National Institute of Health and Care Excellence (NICE) at the first stage of treatment intensification, defined as the “index” drug, between January 2014 and July 2017. Based on previous work we excluded people intensifying treatment with a thiazolidinedione, insulin or a GLP‐1 receptor agonist as these treatments have been infrequently used in recent years and/or fall outside the standard first‐stage guidance.^22^ We excluded women who were pregnant before and after treatment change as guidelines are different for pregnant or breastfeeding women.

To limit the study population to people who intensified rather than changed treatment, we required that 1) a second prescription for the index drug was recorded within 60 days after the end of the first prescription and 2) the individual received a further metformin prescription between the first and second prescription for an intensification drug. We used the date of the first prescription for the first‐stage intensification drug as baseline/study entry.

### 2.3 Outcomes

**Figure 1:**
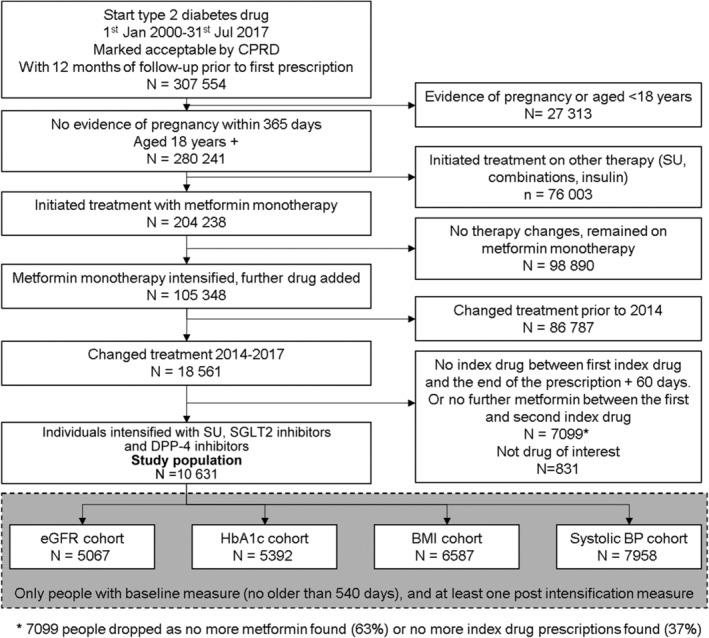
Flow diagram of study participants. BMI, body mass index; BP, blood pressure; CPRD, Clinical Practice Research Datalink; DPP‐4, dipeptidyl peptidase‐4; eGFR, estimated glomerular filtration rate; HbA1c, glycated haemoglobin; SU, sulphonylurea; SGLT2, sodium‐glucose co‐transporter‐2

We chose four clinical measures that are associated with future risk of cardiovascular disease or diabetic complications: glycated haemoglobin (HbA1c); systolic blood pressure (BP); body mass index (BMI); and estimated glomerular filtration rate (eGFR).^27^, ^28^ For each measure we extracted all test results for HbA1c, systolic BP, weight and height to calculate BMI, and serum creatinine to calculate eGFR using the Chronic Kidney Disease Epidemiology Collaboration (CKD‐EPI) equation.^29^ We then created four cohorts which are subsets of the study population for each clinical measure (Figure 1). To be included in a cohort, patients were required to have at least one record of the measure within 540 days prior to drug treatment intensification and at least one follow‐up recording of the variable of interest. Participants in each cohort were followed until the first of: death, leaving the practice, prescription of an alternative drug treatment for type 2 diabetes, or end of study (July 1, 2017).

### 2.4 Descriptive variables and covariates

Details of our cohort methodology have been published previously.^23^ Baseline covariates are those recorded prior to index drug prescription. We only included measurements within 540 days prior to baseline as older values might not reflect the values at the point of treatment intensification. This time point was chosen pragmatically based on the Quality Outcomes Framework recommendation that patients with diabetes have full clinical review annually, with additional time for delays in arranging appointments and for data entry.^30^ Medical diagnoses such as cardiovascular disease and retinopathy were defined as present if they were listed in the medical record on or before the date of drug intensification. We defined use of angiotensin‐converting enzyme inhibitors/angiotensin receptor blockers or statins as any prescription for such a drug in the year before the start of follow‐up.

### 2.5 Statistical analysis

#### 2.5.1 Propensity‐score matching

Variables considered as potential confounders, based on previous work defining factors associated with drug prescription,^23^ were: age; gender; ethnicity; baseline values of HbA1c, eGFR, BMI and systolic BP; baseline diagnosis of cardiovascular disease, retinopathy or current smoking; quintile of IMD score; time taking metformin before intensification; and the year that treatment was intensified.

Propensity‐score matching between the three classes of drugs was used to assemble a sample in which each patient receiving SGLT2 inhibitors was matched to up to four patients prescribed DPP‐4 inhibitors and up to five patients prescribed SUs. These matching goals were chosen to reflect the relative number of users in each group. Each matched set had to include a minimum of one patient from each of the three treatment groups being compared. Patients were matched without replacement on the propensity score within a calliper of 0.025, ~0.2 times the standard deviation of the propensity score. The estimated propensity scores were obtained from logistic regression. An iterative approach to the selection of confounders was taken, including a potential confounder in the model if required to obtain balance of the variable across treatment groups, as measured by the standardized mean difference, accepting imbalances up to 0.2. We matched cohorts on their baseline measures of BMI, systolic BP, eGFR or HbA1c by including additional “exact” matching on each variable. To account for the variability in the number of individuals in the matched sets, patients in incomplete sets were up‐weighted to give each matched set equal weight.^31^ Separate propensity‐score models were fitted to each sub‐cohort (one for each outcome measure). Missing data in confounders were handled using a missing category approach.^32^

#### 2.5.2 Mixed effects linear regression

For each continuous outcome, we applied mixed effects linear regression models to the matched samples, with a random effect for patient, to estimate the mean of the measure over time, for each treatment group. We fitted a cubic model for the outcome over time. Follow‐up time was split at 12, 24, 36, 60, 84 and 96 weeks, with cut‐offs based on commonly reported time periods in clinical trials. Treatment effects were estimated separately in each time band. We used these models to estimate differences in means at 12 and 60 weeks compared to week zero. Overall differences across the 96‐week period were obtained by averaging the period‐specific treatment effect estimates and weighting by the duration of the period. To explore differential drop out over follow‐up, we calculated mean baseline level of HbA1c, eGFR, systolic BP and BMI for all patients remaining in the analysis population at each follow‐up time point.

#### 2.5.3 Sensitivity analyses

To assess the robustness of results to the assumptions made in our primary analysis we completed a series of sensitivity analyses. First, we applied the mixed effects models to 1:1:1 matched samples (rather than matched sets with varying numbers of matches). Second, we removed the censoring when patients were prescribed an additional or alternative diabetic medication, to obtain results analogous to an intention‐to‐treat estimate. Third, we assessed the impact of conducting a complete case analysis by imputing missing data using chained equations. Fourth, we restricted the analysis to patients who had at least one baseline and one follow‐up measure for all four outcome measures, to determine whether the primary results were influenced by inclusion of patients without select measures into different cohorts. Fifth, we excluded individuals from the analysis if they had high numbers of tests for each measure (eGFR, HbA1c, BMI or systolic BP) during follow‐up to assess whether frequent measurements had an impact on the findings.

### 2.6 Patient and public involvement statement

Patients were not involved in the design or conduct of the study. We plan to disseminate the results through peer‐reviewed publication.

### 2.7 Ethics approval

The protocol for this research was approved by the Independent Scientific Advisory Committee of the Medicines and Healthcare products Regulatory Agency Database Research (number 16_267). This study was also approved by the London School of Hygiene and Tropical Medicine Ethics Committee, ref: 11923.

## 3 RESULTS

Within the study population of individuals who intensified from metformin monotherapy with an SU, a DPP‐4 inhibitor or a SGLT2 inhibitor, 40% were women and the mean age, BMI, eGFR and systolic BP were 60 years, 33 kg/m^2^, 89 mL/min/1.73m^2^ and 133 mmHg, respectively (Table 1). The subcohorts for each physiological variable of interest were as follows: eGFR, n = 5067; HbA1c, n = 5392; BMI, n = 6587 and systolic BP, n = 7958. Details of the cohort selection are provided in Figure 1.

**Table 1:** Description of the study population at baseline for individuals intensifying treatment from metformin monotherapy with sulphonylureas, sodium‐glucose co‐transporter‐2 inhibitors or dipeptidyl peptidase‐4 inhibitors between 2014 and 2017

	SUs N = 5010	SGLT2 inhibitors N = 1187	DPP‐4 inhibitors N = 4434
Age, years	61 (13)	55 (10)	61 (12)
Women, n (%)	1988 (39.7)	474 (39.9)	1745 (39.4)
BMI, kg/m^2^	32 (6)	37 (7)	33 (7)
Missing, n (%)	470 (9.4)	54 (4.5)	285 (6.4)
eGFR, mL/min/1.73m^2^	89 (18)	96 (13)	88 (18)
Missing, n (%)	1683 (33.6)	493 (41.5)	1568 (35.4)
Systolic BP, mmHg	133 (14)	134 (14)	133 (14)
Missing, n (%)	837 (16.7)	293 (24.7)	880 (19.8)
HbA1c, mmol/mol	80 (21)	77 (17)	73 (16)
Missing, n (%)	2180 (43.5)	629 (53)	2085 (47)
Metformin treatment, months	40 (37)	36 (33)	44 (37)
Cardiovascular disease, n (%)	707 (14.1)	119 (10)	601 (13.6)
Heart failure, n (%)	194 (3.9)	24 (2)	146 (3.3)
Retinopathy, n (%)	868 (17.3)	181 (15.2)	861 (19.4)
ACE inhibitor/ARB treatment, n (%)	2711 (54.1)	670 (56.4)	2490 (56.2)
Statin treatment, n (%)	3530 (70.5)	819 (69)	3387 (76.4)
IMD, n (%)			
1 (least deprived)	467 (9.3)	93 (7.8)	398 (9.0)
2	485 (9.7)	99 (8.3)	378 (8.5)
3	567 (11.3)	117 (9.9)	449 (10.1)
4	643 (12.8)	99 (8.3)	427 (9.6)
5 (most deprived)	589 (11.8)	81 (6.8)	479 (10.8)
Missing	2259 (45.1)	698 (58.8)	2303 (51.9)
Smoking status, n (%)			
Non‐smoker	1883 (37.6)	462 (38.9)	1642 (37.0)
Current	818 (16.3)	193 (16.3)	688 (15.5)
Ex‐smoker	2297 (45.8)	532 (44.8)	2102 (47.4)
Missing	12 (0.2)	N < 5	N < 5
Ethnicity, n (%)			
White	2052 (41.5)	500 (42.1)	1944 (43.8)
South Asian	229 (4.6)	31 (2.6)	146 (3.3)
Black	122 (2.4)	9 (0.8)	61 (1.4)
Other	59 (1.2)	5 (0.4)	26 (0.6)
Mixed heritage	14 (0.3)	N < 5	16 (0.4)
Missing	2534 (50.6)	640 (53.9)	2241 (50.5)

Abbreviations: ACE, angiotensin‐converting enzyme; ARB, angiotensin 2 receptor blocker; BMI, body mass index; BP, blood pressure; DPP‐4, dipeptidyl peptidase‐4; eGFR, estimated glomerular filtration rate; HbA1c, glycated haemoglobin; IMD, index of multiple deprivation; SGLT2, sodium‐glucose co‐transporter‐2; SU, sulphonylurea.

*Note*: Values for continuous values are mean (SD) and categorical values are n (%), as indicated. % values are of entire cohort. Frequencies below five not stated as per Medicines and Healthcare products Regulatory Agency database research policy.

### 3.1 Propensity‐score matched analysis

Initial imbalances in baseline characteristics across treatment groups were minimized after propensity‐score matching, for each cohort (HbA1c, eGFR, BMI and systolic BP; Figure S1). The propensity scores for SGLT2 inhibitors showed substantial overlap across the three treatment groups (Figure S2).

Table S1 describes the unmatched SGLT2 inhibitor users and Table S2 shows the number of matches identified for each cohort. The proportion of SGLT2 inhibitor users not matched ranged from 3% in the BMI cohort to 11% in the systolic BP cohort. The length of follow‐up (days) and number of repeated measures did not vary substantially between each clinical variable (Table S3).

Table 2 provides the baseline characteristics of the largest propensity‐score matched cohort, that for HbA1c. Baseline characteristics for the eGFR, systolic BP and BMI matched cohorts are shown in Tables S4 to S6. After propensity‐score matching, cohorts were well matched on baseline covariates, and closely matched on the baseline physiological variables of interest. Figure S2 shows the percentage standardized mean difference in baseline covariates for unmatched and matched cohorts, for each measure.

**Table 2:** Description of the propensity‐score matched and weighted glycated haemoglobin cohort at baseline for individuals intensifying treatment from metformin monotherapy with sulphonylureas, sodium‐glucose co‐transporter‐2 inhibitors or dipeptidyl peptidase‐4 inhibitors between 2014 and 2017

	SUs	SGLT2 inhibitors	DPP‐4 inhibitors
Number of individuals^a^	1691	481	1445
Counts after weighting	481	481	481
Age, years	56.4 (11.3)	56.3 (9.6)	56.6 (10.6)
Women, n (%)	191 (40)	191 (40)	190 (39)
BMI, kg/m^2^	34.4 (5.4)	34.8 (5.5)	34.3 (5.4)
eGFR, mL/min/1.73m^2^	93.5 (15.4)	93.3 (12.2)	93.3 (14.7)
Systolic BP, mmHg	133.9 (13.3)	133.7 (12.4)	133.7 (13.2)
HbA1c, mmol/mol	76.7 (18.2)	76.4 (16.8)	76.7 (16.6)
Metformin treatment, months	36.1 (34.4)	38.0 (32.9)	38.2 (35.2)
Cardiovascular disease, n (%)	57 (12)	45 (9)	51 (11)
Heart failure, n (%)	14 (3)	12 (2)	11 (2)
Retinopathy, n (%)	79 (16)	75 (16)	88 (18)
ACE inhibitor/ARB treatment, n (%)	252 (52)	278 (58)	252 (52)
Statin treatment	337 (70)	339 (70)	360 (75)
IMD			
1 (least deprived)	50 (10)	51 (11)	50 (10)
2	51 (11)	54 (11)	51 (11)
3	59 (12)	60 (12)	61 (13)
4	41 (9)	40 (8)	37 (8)
5 (most deprived)	35 (7)	37 (8)	36 (7)
Missing	245 (51)	239 (50)	246 (51)
Smoking status, n (%)			
Non‐smoker	178 (37)	199 (41)	182 (38)
Current	87 (18)	75 (16)	73 (15)
Ex‐smoker	213 (44)	207 (43)	225 (47)
Missing	<5	<5	<5
Ethnicity, n (%)			
White	202 (42)	194 (40)	192 (40)
South Asian	9 (2)	11 (2)	11 (2)
Black	6 (1)	7 (1)	6 (1)
Other	<5	<5	<5
Mixed heritage	<5	<5	<5
Missing	261 (54)	267 (56)	269 (56)

Abbreviations: ACE, angiotensin‐converting enzyme; ARB, angiotensin 2 receptor blocker; BMI, body mass index; BP, blood pressure; DPP‐4, dipeptidyl peptidase‐4; eGFR, estimated glomerular filtration rate; HbA1c, glycated haemoglobin; IMD, Index of multiple deprivation; SU, sulphonylurea; SGLT2, sodium‐glucose co‐transporter‐2.

a Number of individuals contributing data to the HbA1c analysis, before weighting was applied. Values for categorical values are weighted mean (SD) and categorical values are n (%), as indicated, of entire cohort. After iteration of the propensity‐score model, the following covariates were included in the model: age; HbA1c; eGFR; BMI; systolic BP; patient‐level IMD score; and ethnicity. The groups were further matched on decile of baseline HbA1c. Figures provided are weighted means or counts. Frequencies below five not stated as per MHRA Database Research policy.

**Figure 2:**
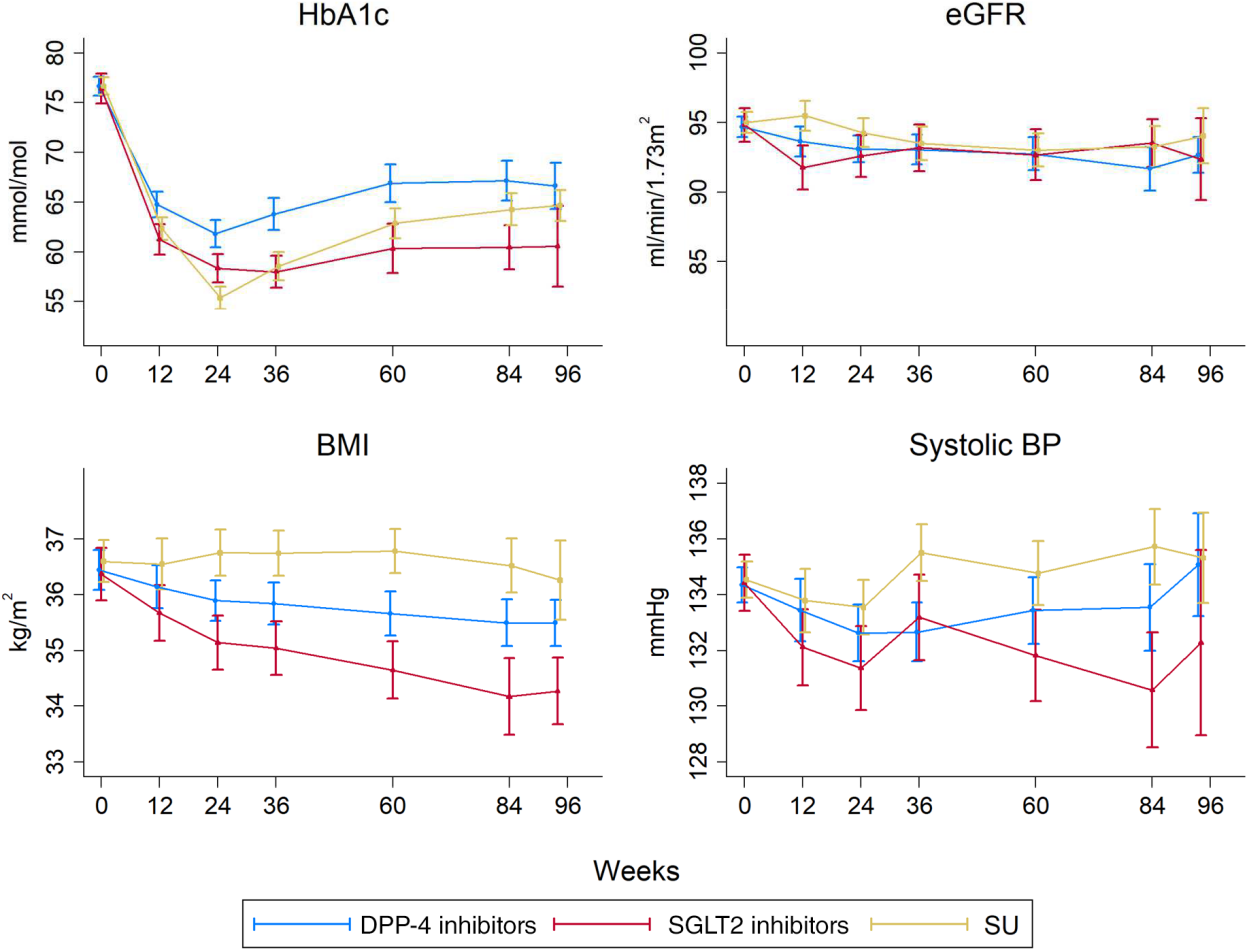
Mean (95% confidence intervals) of each clinical measure during treatment for propensity‐score matched individuals after intensification with a dipeptidyl peptidase‐4 (DPP‐4) inhibitor, a sodium‐glucose co‐transporter‐2 (SGLT2) inhibitor or a sulphonylurea (SU) following metformin monotherapy. BMI, body mass index; BP, blood pressure; eGFR, estimated glomerular filtration rate; HbA1c, glycated haemoglobin

Estimated mean values of each clinical measure for each treatment group at the analysed time points, and changes from baseline, from linear mixed models fitted within the propensity‐score matched cohorts are shown in Figure 2 and Table S7.

HbA1c fell substantially after intensification from a baseline of 76 to 77 mmol/mol for all drugs, but this fall was greatest for SGLT2 inhibitor users. The mean fall at week 12 was −15.2 mmol/mol (95% CI –16.9, −13.5) for SGLT2 inhibitor users, −14.3 mmol/mol (95% CI –15.5, −13.2) for SU users and − 11.9 mmol/mol (95% CI –13.1, −10.6) for DPP‐4 inhibitors users. This fall compared to baseline was similar at 60 weeks of follow‐up for all drug classes. The mean difference over 96 weeks of follow‐up for SGLT2 inhibitor users was −5.4 mmol/mol (95% CI –7.4, −3.4) compared to DPP‐4 inhibitor users and −1.7 (95% CI –3.7, +0.2) compared to SU users.

Baseline systolic BP was 134 to 135 mmHg and fell for SGLT2 inhibitors users throughout follow‐up, but not for DPP‐4 inhibitor or SU users. The mean fall at week 12 was −2.3 mmHg (95% CI –3.8, −0.8) for SGLT2 inhibitor users, −0.8 mmHg (95% CI –1.9, +0.4) for SU users and − 0.9 mmHg (95% CI –2.1, +0.2) for DPP‐4 inhibitor users. At 60 weeks, systolic BP remained lower than baseline for SGLT2 inhibitor users but not for other drug classes. The mean difference over 96 weeks of follow‐up for SGLT2 inhibitor users was −1.82 mmHg (95% CI –3.18, −0.45) compared to DPP‐4 inhibitor users and −3.06 mmHg (95% CI –4.43, −1.68) compared to SU users.

Mean BMI at baseline was 36 to 37 kg/m^2^ and fell compared to baseline over follow‐up for SGLT2 inhibitor and DPP‐4 inhibitor users. The mean fall at week 12 was −0.7 kg/m^2^ (95% CI –0.9, −0.5) for SGLT2 inhibitor users, 0.0 kg/m^2^ (95% CI –0.3, +0.2) for SU users and −0.3 kg/m^2^ (95% CI –0.5, −0.1) for DPP‐4 inhibitor users. At 60 weeks, BMI remained lower than baseline for SGLT2 inhibitor and DPP‐4 inhibitor users but not SU users. These falls in BMI are equivalent to a weight loss of 2.3 kg for a DPP‐4 inhibitor user and 5.0 kg for an SGLT2 inhibitor user at 60 weeks of treatment for a person 1.7 m tall, the mean height of the cohort of patients who were prescribed SGLT2 inhibitors. The mean difference over 96 weeks of follow‐up for SGLT2 inhibitor users was −0.92 kg/m^2^ (95% CI –1.17, −0.66) compared to DPP‐4 inhibitor users and −1.67 kg/m^2^ (95% CI –1.95, −1.38) compared to SU users.

Baseline eGFR was 95 mL/min/1.73m^2^ and fell at 12 weeks for SGLT2 inhibitor and DPP‐4 inhibitor users. The mean fall at week 12 was −3.1 mL/min/1.73m^2^ (95% CI –4.1, −2.0) for SGLT2 inhibitor users, the mean increase was +0.5 mL/min/1.73m^2^ (95% CI –0.4, +1.3) for SU users and the mean fall was −1.0 mL/min/1.73m^2^ (95% CI –1.9, −0.2) for DPP‐4 inhibitor users. At 60 weeks, the fall in eGFR from baseline was ~2 mL/min/1.73m^2^ for each drug class. The mean difference over 96 weeks of follow‐up for SGLT2 inhibitor users was −0.03 mL/min/1.73m^2^ (95% CI –1.01, 0.94) versus DPP‐4 inhibitor users and −0.78 mL/min/1.73m^2^ (95% CI –1.82, −0.27) versus SU users.

During and at the end of follow‐up participants who remained in the cohort were similar in their baseline characteristics to the entire cohort at baseline, suggesting that differential loss to follow‐up of patients whose health status varied importantly from the entire cohort had not occurred (Tables S8–S11).

Results of all sensitivity analyses were all similar to those of the main analysis (Figures S3–S7 and Table S12). The distribution of baseline covariates for individuals excluded because of missing baseline or follow‐up data was similar to that in the study population (Tables S14–S17).

## 4 DISCUSSION

In the present study, we robustly estimated and compared the effects of the three drug options commonly used to intensify metformin monotherapy – SUs, SGLT2 inhibitors and DPP‐4 inhibitors – on HbA1c, BMI, systolic BP and eGFR in UK primary care. In cohorts of people with similar baseline characteristics and levels of each clinical measure we show that all three drug options were associated with large falls in HbA1c, with better overall glycaemic control for people prescribed SGLT2 inhibitors. People prescribed DPP‐4 inhibitors and SGLT2 inhibitors experienced falls in BMI that were sustained over the study duration, with those prescribed SGLT2 inhibitors experiencing about twice the weight loss observed for DPP‐4 inhibitor users. Systolic BP fell compared to baseline at 12 weeks for SGLT2 inhibitor users but not for users of the other drug classes. Over the study duration, systolic BP was ~3 mmHg lower for those prescribed SGLT2 inhibitors compared to those prescribed SUs; however, the CIs for the estimates of systolic BP were large, and overlapped for the SGLT2 inhibitor and DPP‐4 inhibitor cohorts. Users of SGLT2 inhibitors demonstrated falls in eGFR at 12 weeks of treatment but, over time, the fall in eGFR was small and similar for each drug class.

The major strength of the present study is that it reflects recent clinical practice, where relative equipoise about choice of drug class and wide national variation in choice create an opportunity for direct comparison of drug effects. Selecting patients whose drug therapy is being intensified at the same stage of treatment reduces time‐related bias. We have previously examined the differences in characteristics of patients prescribed each drug class in detail and, based on this, have used propensity‐score matching to achieve cohorts of patients very similar in baseline characteristics. Regular monitoring of people with type 2 diabetes in UK primary care provided extensive data, enabling us to use the vast majority of participants from our baseline cohort for modelling each clinical variable.

The relatively short period over which SGLT2 inhibitors have been used in UK primary care, however, means that the sample size was smaller than that of many primary care database studies, with a follow‐up of 2 years, shorter than recent clinical trials. This means that we can only examine class effects and the study would be underpowered to detect drug‐specific effects and endpoints such as cardiovascular disease mortality. We classified the start date of treatment for each intensification drug from the first record in primary care. For a proportion of patients who initiated the drugs in secondary care, this date would be misclassified. Our “baseline” values of physiological variables may therefore have been measured after treatment had started. However, this would have led to underestimation of early differences and, given the short duration of prescriptions issued in secondary care, we would anticipate that this would affect only a very small proportion of our results. Proteinuria data were insufficiently complete to use as a variable in our analysis.

Our study design focused on providing matches of patients prescribed DPP‐4 inhibitors and SUs to patients prescribed SGLT2 inhibitors. This means that the results are generalizable only to contemporary SGLT2 inhibitor users in primary care who had, for example, a high BMI and well preserved renal function compared to users of other drug classes. Patients with a relative contraindication for a drug, for example, those with poor renal function (and therefore prescribed DPP‐4 inhibitors or SUs), would not have been matched. Nonetheless, this study design does provide a robust comparison of the drug effects in routine care for patients for whom there was the possibility of being prescribed one of the three drug classes.

Finally, we sought to study the biological effects of the drug classes, therefore, we censored follow‐up when patients commenced treatment with an alternative drug class, analogous to an “as‐treated” analysis in a clinical trial. If a greater proportion of patients stopped treatment with one of the drug classes this would limit the validity of between‐drug comparisons, particularly if the decision to stop treatment was associated with an outcome variable (such as failure for glycaemic control to improve). However, we saw similar results in our simulated “intention‐to‐treat” analysis, where we did not censor patients when they changed treatment, suggesting that this has not substantially impacted our results. As a small proportion of the cohort (4%) stop the initial drug and do not restart a different diabetic treatment (which would lead to censoring), clinical measures early on in the study period are likely to most closely represent the “as‐treated” drug effects.

As we have shown previously, SGLT2 inhibitors are prescribed to a different population in UK primary care compared to patients enrolled in recent major outcome trials (Table S13).^23^ Participants in our study were younger, with better renal function, and included a lower proportion of people with cardiovascular disease, heart failure and retinopathy. Our study population had poorer glycaemic control and was heavier at baseline compared to participants in recent cardiovascular outcome studies. Perhaps related to this, our study participants also showed greater improvement after initiating SGLT2 inhibitors compared to trial participants. We found a fall in HbA1c equivalent to 1.4% after 12 weeks of treatment, while clinical trial HbA1c fall estimates range from −0.25% (95% CI –0.31, −0.20) in CREDENCE to −0.58% (95% CI 0.61, −0.56) in CANVAS.

For patients commencing SGLT2 inhibitors, the present study estimated falls in BMI compared to baseline equivalent to weight loss of 2 kg at 12 weeks for an individual 1.7 m tall. Outcome studies show weight loss ranging from 1 kg at 12 weeks in CREDENCE to 2 kg at 6 months in DECLARE‐TIMI. At the end of the present study, mean weight loss compared to baseline was 5 kg for SGLT2 inhibitor users compared to 2 kg in CREDENCE and 4 kg in DECLARE‐TIMI.

Falls in BP and eGFR on initiating treatment with SGLT2 inhibitors are widely recognized and, in the present study, we found striking similarities between the effects seen in clinical trials and in our routine care population, although there is substantial uncertainty around our estimates. We found a mean fall in systolic BP of 2.3 mmHg (95% CI –3.8, −0.8) compared to baseline at 12 weeks for SGLT2 inhibitor users, but no fall for those prescribed other drug classes. Trial falls in systolic BP compared to baseline range from 2.8 mmHg at 12 weeks in the CREDENCE study to 5.5 mmHg in EMPA‐REG (10‐mg dose arm). Over the duration of the study, our results showed a mean difference in systolic BP of −3.06 mmHg (95% CI –4.43, −1.68) compared to SU‐treated patients. Estimates compared to placebo in clinical trials were very similar, ranging from −2.7 mmHg (95% CI –3.0, −2.4) in the DECLARE‐TIMI study to −3.93 mmHg (95% CI –4.30, −3.56) in CANVAS.

For renal function we found a fall in eGFR of −3.1 mL/min/1.73 m^2^ (95% CI –4.1, −2.0) at 12 weeks, similar to that observed at 3 weeks (−3.72 ± 0.25 mL/min/1.73 m^2^) in CREDENCE and the same as that observed in CANVAS at 12 weeks (−3.1 ± 0.1 mL/min/1.73 m^2^). At 60 weeks we saw a fall of −2.2 mL/min/1.73 m^2^ (95% CI –3.6, −0.7), again, very similar to estimates reported in clinical trials, for example, a slope of 2.74 mL/min/1.73 m^2^ per year (95% CI 2.37, 3.11) in CREDENCE. However, unlike the clinical trials, falls in eGFR in our comparison group were not different from those in SGLT2 inhibitor‐treated patients, ~2 mL/min/1.73 m^2^ at 60 weeks for patients treated with SUs and DPP‐4 inhibitors. By contrast placebo‐treated patients in CREDENCE had a slope of decline of renal function of −4.59 mL/min/1.73 m^2^ per year, while in CANVAS they had a difference from baseline of −3.9 ± 0.2 mL/min/1.73 m^2^ at a mean follow‐up of 188 weeks.

These results demonstrate the huge value of primary care data for conducting observational research. Estimates for both improvement in glycaemic control and HbA1c were very similar to those found in previous research on intensification of treatment for type 2 diabetes using the CPRD,^19^ which provides validation of our methods. This is the first study to examine how changes in BP and renal function relate to changes observed in clinical trials using CPRD data. Given the consistency of the results, we are reassured that the benefits of SGLT2 inhibitors seen in clinical trials will be maintained in routine care, although given the lower risk profile of SGLT2 inhibitor‐treated patients, evidence of hard outcome benefits may take longer to accrue. This is particularly the case for outcomes related to renal function, where our results suggest that the rate of renal decline is slower in non‐SGLT2‐inhibitor‐treated patients than that observed in clinical trials, which may reflect the overall lower risk profile (younger with higher baseline eGFR) or the tighter glycaemic control seen in patients treated with other active agents in routine care.

In conclusion, routine primary care data can be used to study the effect of the new classes of treatments for type 2 diabetes on a range of biological variables, and provide estimates that are directly comparable to those seen in controlled clinical trials. Although SGLT2 inhibitor use was associated with the largest reductions in glycaemic control, weight and blood pressure, SUs and DPP‐4 inhibitors were also associated with beneficial changes, reinforcing the need for active comparator outcome trials of these drugs.

## CONFLICTS OF INTEREST

H.S.‐F. is an employee of and holds shares in GSK. I.J.D. is funded by, holds stock in and has consulted for GSK. D.F. has consulted for clinical trial adjudication associated with oral hypoglycaemia medications (ACI clinical), and consulted for Boehinger‐Ingelheim. A.P. reports personal fees from NovoNordisk, Boehringer Ingleheim and Lilly, outside of the submitted work. L.S. has received grants from GSK and from the Wellcome trust, the MRC, the National Institute for Health Research, the British Heart Foundation and Diabetes UK, outside of the submitted work, and is a Trustee of the British Heart Foundation. L.A.T. and E.W. have no relevant conflicts of interest to disclose.

## AUTHOR CONTRIBUTIONS

S.W., L.A.T., I.D., H.S.‐F., E.W. and L.S. conceived and devised the study. E.W. and S.W. analysed the data. All authors contributed to the interpretation of the data. S.W. drafted the article and all authors reviewed and edited the manuscript, and approved the version to be published. The corresponding author attests that all listed authors meet authorship criteria and that no others meeting the criteria have been omitted. L.A.T. is the guarantor for the work and accepts full responsibility for the work and the conduct of the study, had access to the data, and controlled the decision to publish.

## Supporting information


**Appendix** **S1**: Supporting Information

## Data Availability

All codes used in this analysis are available on the Electronic Health Records Research Group Data Compass website: http://datacompass.lshtm.ac.uk/692/. Due to CPRD licence restrictions, no further data sharing is available.
