# Supplementary material for: Comparative effects of sulphonylureas, dipeptidyl peptidase‐4 inhibitors and sodium‐glucose co‐transporter‐2 inhibitors added to metformin monotherapy: a propensity‐score matched cohort study in UK primary care
Source: Diabetes Obes Metab. 2020 Feb 13;22(5):847–56. doi: 10.1111/dom.13970 (PMC7187358; doi:10.1111/dom.13970)
Supplement: Supplementary file 1 — Appendix S1: Supporting Information [file DOM-22-847-s001.docx]

Supplementary Material for “Comparative effects of sulfonylureas, DPP4is and SGLT2 inhibitors added to metformin monotherapy: a propensity-score matched cohort study in UK primary care”


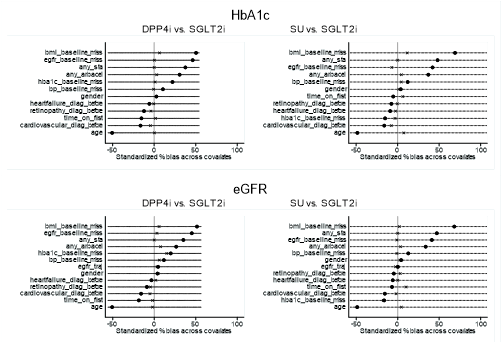


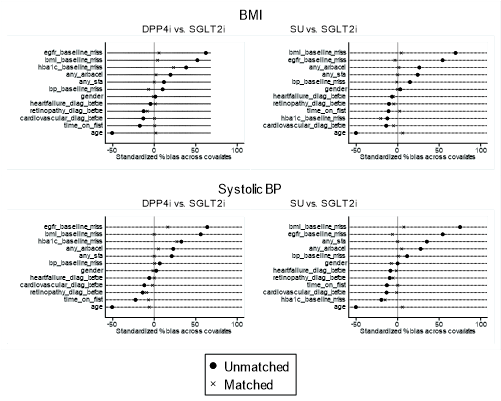


***Supplementary Figure 1: Standardised mean % differences in baseline characteristics for each cohort, unadjusted and compared to the final selected weighted matched sample***

*Where hba1c_baseline_miss, egfr_baseline_miss, bmi_baseline_miss and bp_baseline_miss are the baseline measures, after accounting for missingness in the variables. egfr_traj: eGFR trajectory, time_on_first: time taking metformin prior to changing drug, cardiovascular_diag_before and retinopathy_diag_before are indicators of cardiovascular disease and retinopathy respectively. any_arb and any_sta are indicators of prescriptions for ARB or statins prior to baseline. imd_pt: patient-level index of multiple deprivation*


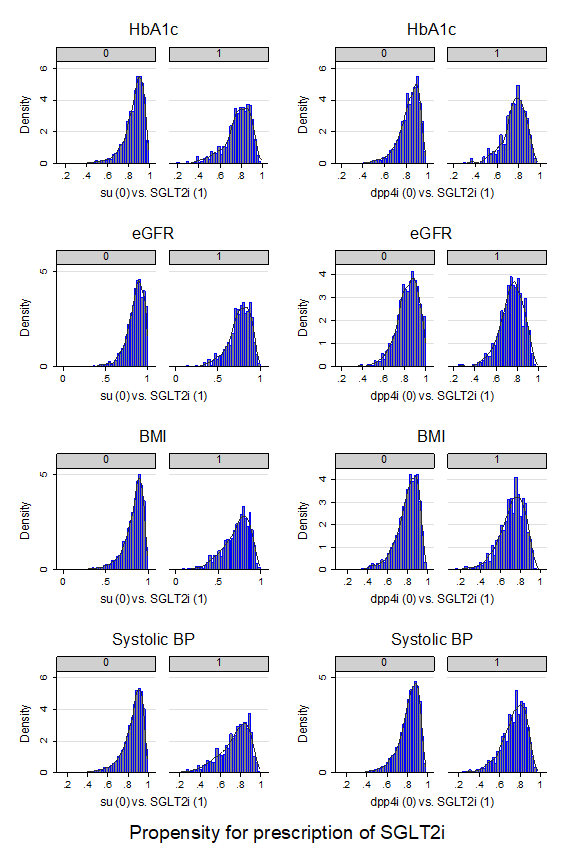


***Supplementary Figure 2: Propensity score for SGLT2i for each measure of interest, in final selected PS model.***

| Measure | Number of SGLT2is available | Number of unmatched SGLT2is | Mean of measure in unmatched | DPP4i contrast | SU contrast |
| --- | --- | --- | --- | --- | --- |
|  |  |  |  | PS for SGLT2i  Mean (SD) | PS for SGLT2i  Mean (SD) |
| eGFR | 483 | 21 | 94.0 | 0.52 (0.12) | 0.45 (0.13) |
| HbA1c | 516 | 35 | 75.2 | 0.54 (0.11) | 0.49 (0.11) |
| BMI | 764 | 26 | 43.9 | 0.47 (0.15) | 0.45 (0.17) |
| Sys BP | 824 | 88 | 136.1 | 0.54 (0.11) | 0.49 (0.14) |

**Supplementary Table 1: Description of unmatched SGLT2is**

| Measure |  | Mean number of SU matches | Mean number of DPP4i matches |
| --- | --- | --- | --- |
| eGFR |  | 3.53 | 2.97 |
| HbA1c |  | 3.52 | 3.00 |
| BMI |  | 2.92 | 2.66 |
| Sys BP |  | 3.47 | 2.97 |

**Supplementary Table 2: Number of matches found for each treatment group (Aim was for 5:1:4 SU:SGLT2i:DPP4i)**

|  | Length of follow-up | Mean number of measures for analysis cohort | | |
| --- | --- | --- | --- | --- |
|  | Days, mean (median) | DPP4i | SGLT2i | SU |
| eGFR | 616 (582) | 5.5 | 5.4 | 6.1 |
| HbA1c | 605 (572) | 5.5 | 5.1 | 5.8 |
| BMI | 624 (590) | 5.4 | 5.6 | 5.5 |
| Systolic BP | 617 (582) | 7.1 | 6.5 | 7.5 |

**Supplementary Table 3: Length of follow-up (days) and number of repeated measures available for each analysis cohort**

|  | **Category** | **SU** | **SGLT2i** | **DPP4i** |
| --- | --- | --- | --- | --- |
| **Counts** |  | 1630 | 462 | 1374 |
| **Age at baseline** | Years | 55.8 (10.9) | 56 (10.3) | 56.4 (10.8) |
| **BMI baseline** | kg/m^2^ | 34.9 (5.9) | 35 (5.7) | 34.9 (5.7) |
| **eGFR baseline** | ml/min/1.73m^2^ | 95 (13.8) | 94.8 (13.2) | 94.7 (13.6) |
| **Systolic BP at baseline** | mmHg | 133.8 (13.3) | 134.1 (12.6) | 133.8 (13.3) |
| **HbA1c at baseline** | mmol/mol | 76.1 (17.6) | 75.9 (16.1) | 76 (16.2) |
| **Time taking metformin prior to intensification** | Month | 36.1 (34.8) | 37.7 (32.6) | 38.1 (34.4) |
| **Gender** | Female, n (%) | 179 (39) | 189 (41) | 179 (39) |
| **Diagnosis of CVD** | n (%) | 51 (11) | 45 (10) | 45 (10) |
| **Diagnosis of HF** | n (%) | 15 (3) | 14 (3) | 10 (2) |
| **Diagnosis of retinopathy** | n (%) | 75 (16) | 75 (16) | 88 (19) |
| **Prescription for ARB or ACE inhibitor** | n (%) | 243 (53) | 273 (59) | 246 (53) |
| **Prescription for statin** | n (%) | 319 (69) | 324 (70) | 343 (74) |
| **Patient-level index of multiple deprivation** | 1 LEAST deprived | 47 (10) | 48 (10) | 47 (10) |
|  | 2 | 45 (10) | 47 (10) | 43 (9) |
|  | 3 | 60 (13) | 58 (13) | 59 (13) |
|  | 4 | 43 (9) | 41 (9) | 40 (9) |
|  | 5 MOST deprived | 40 (9) | 32 (7) | 37 (8) |
|  | Missing category | 228 (49) | 236 (51) | 237 (51) |
| **Smoking status** | Non-smoker | 180 (39) | 178 (39) | 178 (39) |
|  | Current | 80 (17) | 72 (16) | 69 (15) |
|  | Ex-smoker | 200 (43) | 212 (46) | 215 (47) |
|  | Missing category | <5 | <5 | <5 |
| **Ethnicity** | White | 191 (41) | 193 (42) | 189 (41) |
| n (%) | South Asian | 12 (3) | 10 (2) | 9 (2) |
|  | Black | 8 (2) | 7 (2) | 7 (2) |
|  | Other | 1 (0) | 1 (0) | 0 (0) |
|  | Mixed | 1 (0) | 2 (0) | 1 (0) |
|  | Missing category | 249 (54) | 249 (54) | 255 (55) |

**Supplementary Table 4: Baseline characteristics for final weighted matched sample for eGFR**After iteration of the propensity score model, the following covariates were included in the model: age, HbA1c, eGFR, eGFR^2^, BMI, systolic BP, patient-level IMD, ethnicity. The group was further matched on quintiles of baseline eGFR % is of entire cohort.

|  | **Category** | | | **SU** | | **SGLT2i** | | **DPP4i** | |
| --- | --- | --- | --- | --- | --- | --- | --- | --- | --- |
| **Count** | |  | 2158 | | 738 | | 1960 | |  |
| **Age at baseline** | Years | | | 55.6 (11.5) | | 55.2 (10.1) | | 56.1 (10.6) | |
| **BMI baseline** | kg/m^2^ | | | 36.6 (6.2) | | 36.4 (6.5) | | 36.4 (6.2) | |
| **eGFR baseline** | ml/min/1.73m^2^ | | | 92.8 (12.8) | | 92.6 (9.9) | | 92.1 (11.2) | |
| **Systolic BP at baseline** | mmHg | | | 134.1 (13.5) | | 134 (13.4) | | 134 (13.1) | |
| **HbA1c at baseline** | mmol/mol | | | 76.8 (14.1) | | 76.5 (12.4) | | 75.9 (11.8) | |
| **Time taking metformin prior to intensification** | Month | | | 36.2 (35.1) | | 36.4 (33.2) | | 37.1 (34.2) | |
| **Gender** | Female,  n (%) | | | 325 (44) | | 303 (41) | | 316 (43) | |
| **Diagnosis of CVD** | n (%) | | | 85 (11) | | 73 (10) | | 77 (10) | |
| **Diagnosis of HF** | n (%) | | | 20 (3) | | 15 (2) | | 17 (2) | |
| **Diagnosis of retinopathy** | n (%) | | | 127 (17) | | 111 (15) | | 123 (17) | |
| **Prescription for ARB or ACE inhibitor** | n (%) | | | 412 (56) | | 419 (57) | | 408 (55) | |
| **Prescription for statin** | n (%) | | | 505 (68) | | 516 (70) | | 559 (76) | |
| **Patient-level index of multiple deprivation** | 1 LEAST deprived | | | 57 (8) | | 60 (8) | | 58 (8) | |
|  | 2 | | | 60 (8) | | 57 (8) | | 58 (8) | |
|  | 3 | | | 76 (10) | | 72 (10) | | 70 (10) | |
|  | 4 | | | 69 (9) | | 58 (8) | | 65 (9) | |
|  | 5 MOST deprived | | | 54 (7) | | 48 (7) | | 45 (6) | |
|  | Missing category | | | 421 (57) | | 443 (60) | | 442 (60) | |
| **Smoking status** | Non-smoker | | | 285 (39) | | 284 (38) | | 279 (38) | |
|  | Current | | | 129 (18) | | 113 (15) | | 124 (17) | |
|  | Ex-smoker | | | 322 (44) | | 341 (46) | | 334 (45) | |
|  | Missing category | | | <5 | | <5 | | <5 | |
| **Ethnicity** | White | | | 313 (42) | | 306 (41) | | 310 (42) | |
| n (%) | South Asian | | | 21 (3) | | 19 (3) | | 14 (2) | |
|  | Black | | | 8 (1) | | 6 (1) | | 7 (1) | |
|  | Other | | | 2 (0) | | 2 (0) | | 2 (0) | |
|  | Mixed | | | 2 (0) | | 2 (0) | | 2 (0) | |
|  | Missing category | | | 392 (53) | | 403 (55) | | 403 (55) | |

**Supplementary Table 5: Baseline characteristics for final weighted matched sample for BMI**

After iteration of the propensity score model, the following covariates were included in the model: age, HbA1c, eGFR, BMI, BMI^2^, systolic BP, patient-level IMD, ethnicity. The groups were further matched on centiles of baseline BMI. % is of entire cohort.

|  | **Category** | **SU** | **SGLT2i** | **DPP4i** |
| --- | --- | --- | --- | --- |
| **Counts** |  | 2554 | 736 | 2186 |
| **Age at baseline** | Years | 56.6 (11.5) | 56.5 (9.8) | 56.5 (10.6) |
| **BMI baseline** | kg/m^2^ | 34.7 (5.5) | 35.1 (5.5) | 34.7 (5.4) |
| **eGFR baseline** | ml/min/1.73m^2^ | 91.6 (12.7) | 91.5 (9.6) | 91.2 (11) |
| **Systolic BP at baseline** | mmHg | 134.5 (14.4) | 134.4 (14) | 134.4 (14.1) |
| **HbA1c at baseline** | mmol/mol | 76 (13) | 75.8 (12.1) | 75.5 (11.4) |
| **Time taking metformin prior to intensification** | Month | 37.2 (35.3) | 36.5 (32.4) | 38.1 (34.3) |
| **Gender** | Female, n (%) | 305 (41) | 295 (40) | 283 (38) |
| **Diagnosis of CVD** | n (%) | 92 (13) | 80 (11) | 82 (11) |
| **Diagnosis of HF** | n (%) | 21 (3) | 17 (2) | 17 (2) |
| **Diagnosis of retinopathy** | n (%) | 119 (16) | 107 (15) | 128 (17) |
| **Prescription for ARB or ACE inhibitor** | n (%) | 414 (56) | 438 (60) | 407 (55) |
| **Prescription for statin** | n (%) | 514 (70) | 517 (70) | 555 (75) |
| **Patient-level index of multiple deprivation** | 1 LEAST deprived | 65 (9) | 64 (9) | 59 (8) |
|  | 2 | 59 (8) | 56 (8) | 55 (7) |
|  | 3 | 75 (10) | 76 (10) | 76 (10) |
|  | 4 | 60 (8) | 60 (8) | 59 (8) |
|  | 5 MOST deprived | 53 (7) | 55 (7) | 54 (7) |
|  | Missing category | 425 (58) | 425 (58) | 433 (59) |
| **Smoking status** | Non-smoker | 282 (38) | 293 (40) | 276 (37) |
|  | Current | 125 (17) | 113 (15) | 118 (16) |
|  | Ex-smoker | 327 (44) | 330 (45) | 342 (47) |
|  | Missing category | <5 | <5 | <5 |
| **Ethnicity** | White | 298 (41) | 291 (40) | 295 (40) |
| n (%) | South Asian | 16 (2) | 19 (3) | 17 (2) |
|  | Black | 9 (1) | 9 (1) | 9 (1) |
|  | Other | <5 | <5 | <5 |
|  | Mixed | <5 | <5 | <5 |
|  | Missing category | 410 (56) | 412 (56) | 410 (56) |

**Supplementary Table 6: Baseline characteristics for final weighted matched sample for Systolic BP**

After iteration of the propensity score model, the following covariates were included in the model: age, HbA1c, eGFR, BMI, systolic BP, patient-level IMD, ethnicity. The groups were further matched on 20 groups of baseline systolic BP. % is of entire cohort.

|  |  | **HbA1c (*mmol/mol)*** | | | ***eGFR (ml/min/1.73m^2^)*** | | |
| --- | --- | --- | --- | --- | --- | --- | --- |
|  | **Week:** | **0** | **12** | **60** | **0** | **12** | **60** |
| **SU** | **Absolute value** | 76.7  (75.8-77.5) | 62.3  (61.2-63.5) | 62.9  (61.4-64.4) | 95.0  (94.3-95.8) | 95.5  (94.4-96.5) | 93.0  (91.8-94.2) |
|  | **Change from baseline** |  | -14.3  (-15.5, -13.2) | -13.8  (-15.4, -12.2) |  | 0.5  (-0.4, 1.3) | -2.0  (-2.9, -1.1) |
| **SGLT2i** | **Absolute value** | 76.4  (74.9-77.9) | 61.2  (59.7-62.8) | 60.3  (57.9-62.8) | 94.8  (93.6-96.0) | 91.8  (90.2-93.3) | 92.7  (90.9-94.5) |
|  | **Change from baseline** |  | -15.2  (-16.9, -13.5) | -16.1  (-18.7, -13.5) |  | -3.1  (-4.1, -2.0) | -2.2  (-3.6, -0.7) |
| **DPP4i** | **Absolute value** | 76.7  (75.7-77.6) | 64.8  (63.5-66.1) | 66.9  (65.0-68.8) | 94.7  (93.9-95.4) | 93.6  (92.6-94.7) | 92.8  (91.6-93.9) |
|  | **Change from baseline** |  | -11.9  (-13.1, -10.6) | -9.8  (-11.6, -7.9) |  | -1.0  (-1.9, -0.2) | -1.9  (-2.9, -1.0) |

|  |  | ***BMI (kg/m^2^)*** | | | ***Systolic BP (mmHg)*** | | |
| --- | --- | --- | --- | --- | --- | --- | --- |
|  | **Week:** | **0** | **12** | **60** | **0** | **12** | **60** |
| **SU** | **Absolute value** | 36.6  (36.2-37.0) | 36.6  (36.1-37.0) | 36.8  (36.4-37.2) | 134.5  (133.9-135.2) | 133.8  (132.7-134.9) | 134.8  (133.6-135.9) |
|  | **Change from baseline** |  | 0.0  (-0.3, 0.2) | 0.2  (0.0, 0.4) |  | -0.8 (-1.9, -0.4) | 0.2  (-1.0, 1.4) |
| **SGLT2i** | **Absolute value** | 36.4  (35.9-36.8) | 35.7  (35.2-36.2) | 34.6  (34.1-35.2) | 134.4  (133.4-135.4) | 132.1  (130.7-133.5) | 131.8  (130.2-133.5) |
|  | **Change from baseline** |  | -0.7  (-0.9, -0.5) | -1.7  (-2.1, -1.4) |  | -2.3  (-3.8, -0.8) | -2.6  (-4.4, -0.8) |
| **DPP4i** | **Absolute value** | 36.4  (36.1-36.8) | 36.1  (35.8-36.5) | 35.7  (35.3-36.1) | 134.4  (133.7-135.0) | 133.4  (132.3-134.6) | 133.4  (132.2-134.6) |
|  | **Change from baseline** |  | -0.3  (-0.5, -0.1) | -0.8  (-1.0, -0.6) |  | -0.9  (-2.1, 0.2) | -0.9  (-2.1, 0.3) |

**Supplementary Table 7. Mean (95% confidence intervals) absolute values and change from baseline for clinical variables at baseline, 12, and 60 weeks, for propensity score matched cohorts of individuals following intensification with DPP4i, SGLT2i and SU after metformin monotherapy.** Abbreviations: SU: Sulfonylurea, DPP4i: dipeptidyl peptidase 4 inhibitors, SGLT2i: Sodium-glucose co-transporter-2 inhibitors, HbA1c: Haemoglobin A1c, eGFR: estimated glomerular filtration rate, BMI: Body mass index, BP: Blood pressure.

|  | Mean HbA1c | N | Mean HbA1c | N | Mean HbA1c | N | Mean HbA1c | N | Mean HbA1c | N | Mean HbA1c | N | Mean HbA1c | N |
| --- | --- | --- | --- | --- | --- | --- | --- | --- | --- | --- | --- | --- | --- | --- |
| Week: | 0 | 0 | 12 | 12 | 24 | 24 | 36 | 36 | 48 | 48 | 60 | 60 | 72 | 72 |
| DPP4i | 76.66 | 1445 | 76.35 | 1396 | 75.64 | 1271 | 75.36 | 1122 | 75.32 | 991 | 75.00 | 868 | 74.81 | 740 |
| SGLT2i | 76.43 | 481 | 76.33 | 470 | 76.24 | 409 | 76.27 | 355 | 76.69 | 305 | 77.19 | 260 | 77.09 | 213 |
| SU | 76.66 | 1691 | 76.55 | 1657 | 76.25 | 1558 | 76.17 | 1443 | 75.94 | 1330 | 75.65 | 1204 | 75.65 | 1071 |

**Supplementary Table 8: Impact of attrition through patients leaving the cohort on HbA1c (mmol/mol). At each time point the mean values represent the BASELINE weighted mean values of HbA1c for participants (and numbers) still contributing data over time for the HbA1c cohort.**

|  | Mean eGFR | N | Mean eGFR | N | Mean eGFR | N | Mean eGFR | N | Mean eGFR | N | Mean eGFR | N | Mean eGFR | N |
| --- | --- | --- | --- | --- | --- | --- | --- | --- | --- | --- | --- | --- | --- | --- |
| Week: | 0 | 0 | 12 | 12 | 24 | 24 | 36 | 36 | 48 | 48 | 60 | 60 | 72 | 72 |
| DPP4i | 94.68 | 1374 | 94.59 | 1334 | 94.56 | 1228 | 94.65 | 1087 | 94.36 | 978 | 94.56 | 853 | 94.31 | 728 |
| SGLT2i | 94.83 | 462 | 94.94 | 456 | 95.23 | 410 | 95.24 | 362 | 95.21 | 310 | 95.51 | 266 | 95.58 | 223 |
| SU | 95.00 | 1630 | 95.07 | 1608 | 95.09 | 1523 | 95.03 | 1414 | 94.97 | 1315 | 94.95 | 1175 | 94.60 | 1034 |

**Supplementary Table 9: Impact of attrition through patients leaving the cohort on eGFR (ml/min/1.73m^2^). At each time point the mean values represent the BASELINE weighted mean values of eGFR for participants (and numbers) still contributing data over time for the eGFR cohort.**

|  | Mean BMI | N | Mean BMI | N | Mean BMI | N | Mean BMI | N | Mean BMI | N | Mean BMI | N | Mean BMI | N |
| --- | --- | --- | --- | --- | --- | --- | --- | --- | --- | --- | --- | --- | --- | --- |
| Week | 0 | 0 | 12 | 12 | 24 | 24 | 36 | 36 | 48 | 48 | 60 | 60 | 72 | 72 |
| DPP4i | 36.45 | 1960 | 36.40 | 1921 | 36.33 | 1763 | 36.29 | 1610 | 36.14 | 1442 | 35.98 | 1242 | 36.07 | 1041 |
| SGLT2i | 36.37 | 738 | 36.33 | 715 | 36.30 | 638 | 36.18 | 558 | 36.15 | 489 | 36.22 | 427 | 36.51 | 348 |
| SU | 36.60 | 2158 | 36.57 | 2117 | 36.56 | 2006 | 36.50 | 1883 | 36.45 | 1763 | 36.40 | 1580 | 36.28 | 1419 |

**Supplementary Table 10: Impact of attrition through patients leaving the cohort on BMI (kg/m^2^). At each time point the mean values represent the BASELINE weighted mean values of BMI for participants (and numbers) still contributing data over time for the BMI cohort.**

|  | Mean BP | N | Mean BP | N | Mean BP | N | Mean BP | N | Mean BP | N | Mean BP | N | Mean BP | N |
| --- | --- | --- | --- | --- | --- | --- | --- | --- | --- | --- | --- | --- | --- | --- |
| Week | 0 | 0 | 12 | 12 | 24 | 24 | 36 | 36 | 48 | 48 | 60 | 60 | 72 | 72 |
| DPP4i | 134.4 | 2186 | 134.2 | 2134 | 134.0 | 1962 | 133.9 | 1771 | 134.2 | 1577 | 134.0 | 1355 | 133.8 | 1143 |
| SGLT2i | 134.4 | 736 | 134.3 | 718 | 134.1 | 641 | 133.7 | 562 | 133.8 | 487 | 133.8 | 427 | 133.5 | 345 |
| SU | 134.5 | 2554 | 134.4 | 2496 | 134.3 | 2352 | 134.3 | 2190 | 134.5 | 2042 | 134.5 | 1840 | 134.4 | 1634 |

**Supplementary Table 11: Impact of attrition through patients leaving the cohort on systolic BP (mmHg). At each time point the mean values represent the BASELINE weighted mean values of systolic BP for participants (and numbers) still contributing data over time for the systolic BP cohort.**

**
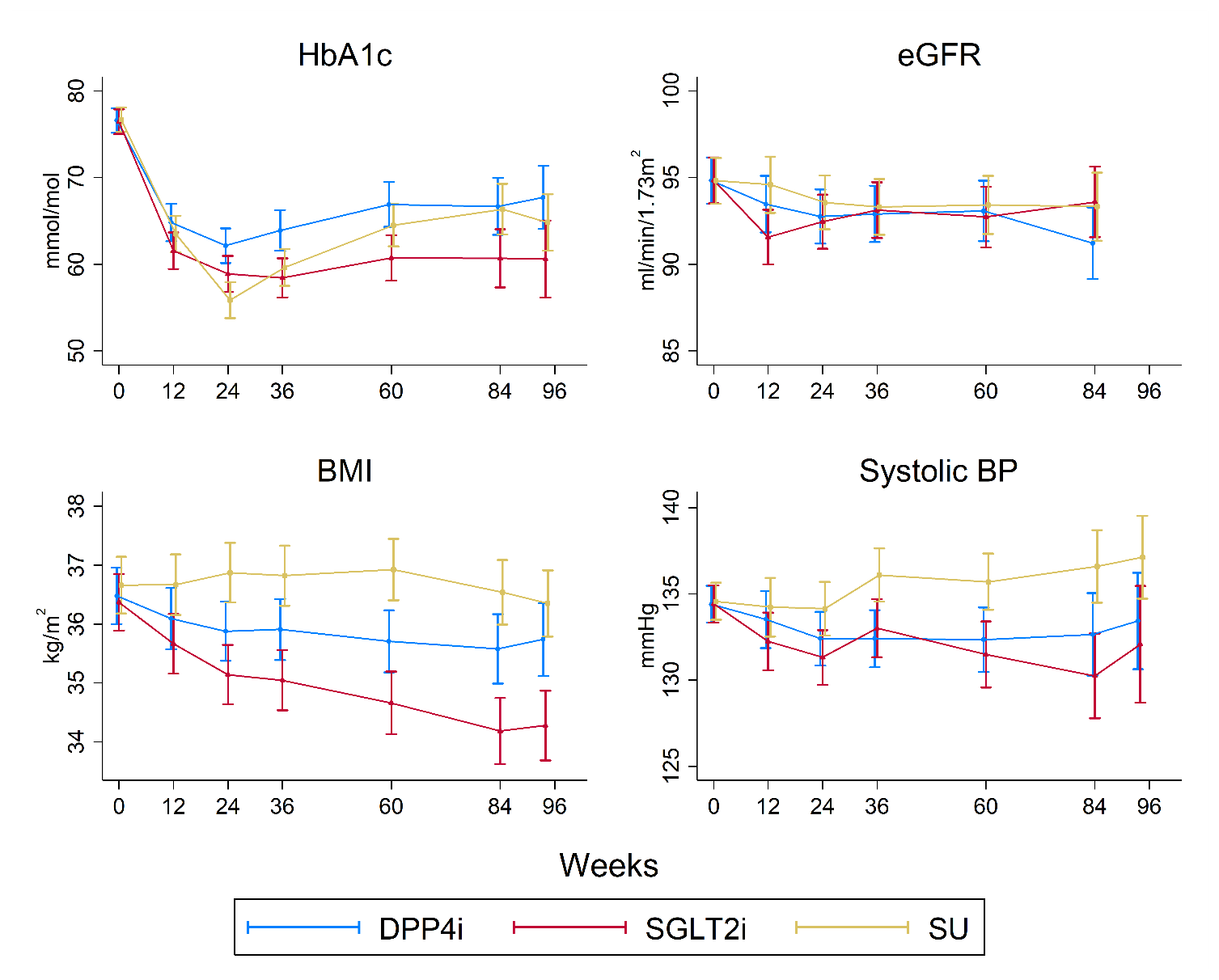
**

**Supplementary Figure 3: Mean (95% confidence intervals) of each clinical measure during treatment for 1:1:1 matched propensity score matched cohorts of individuals following intensification with DPP4i, SGLT2i and SU after metformin monotherapy**


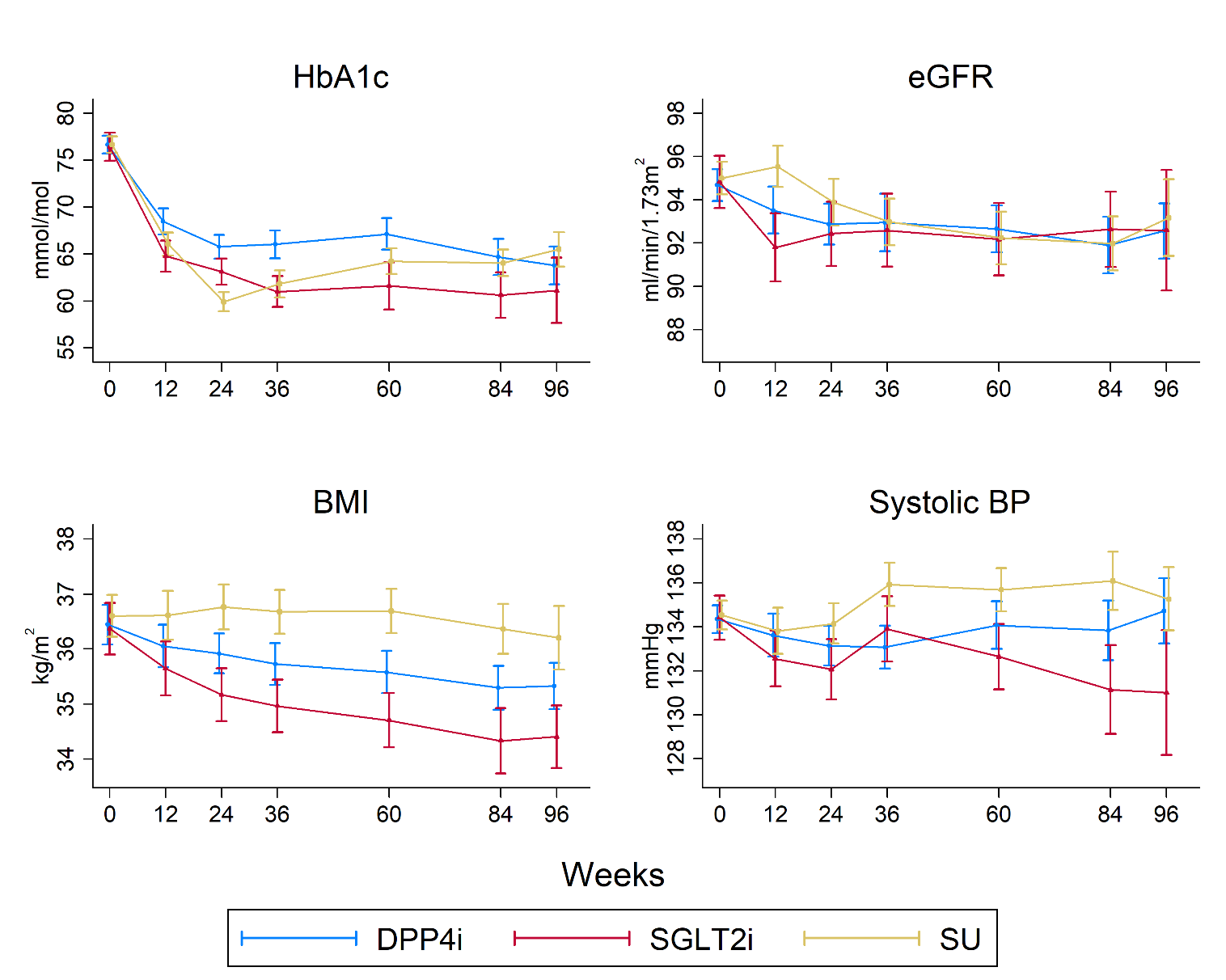


**Supplementary Figure 4: Mean (95% confidence intervals) of each clinical measure during treatment, for propensity score matched cohorts of individuals (intention to treat model), following intensification with DPP4i, SGLT2i and SU after metformin monotherapy**

**
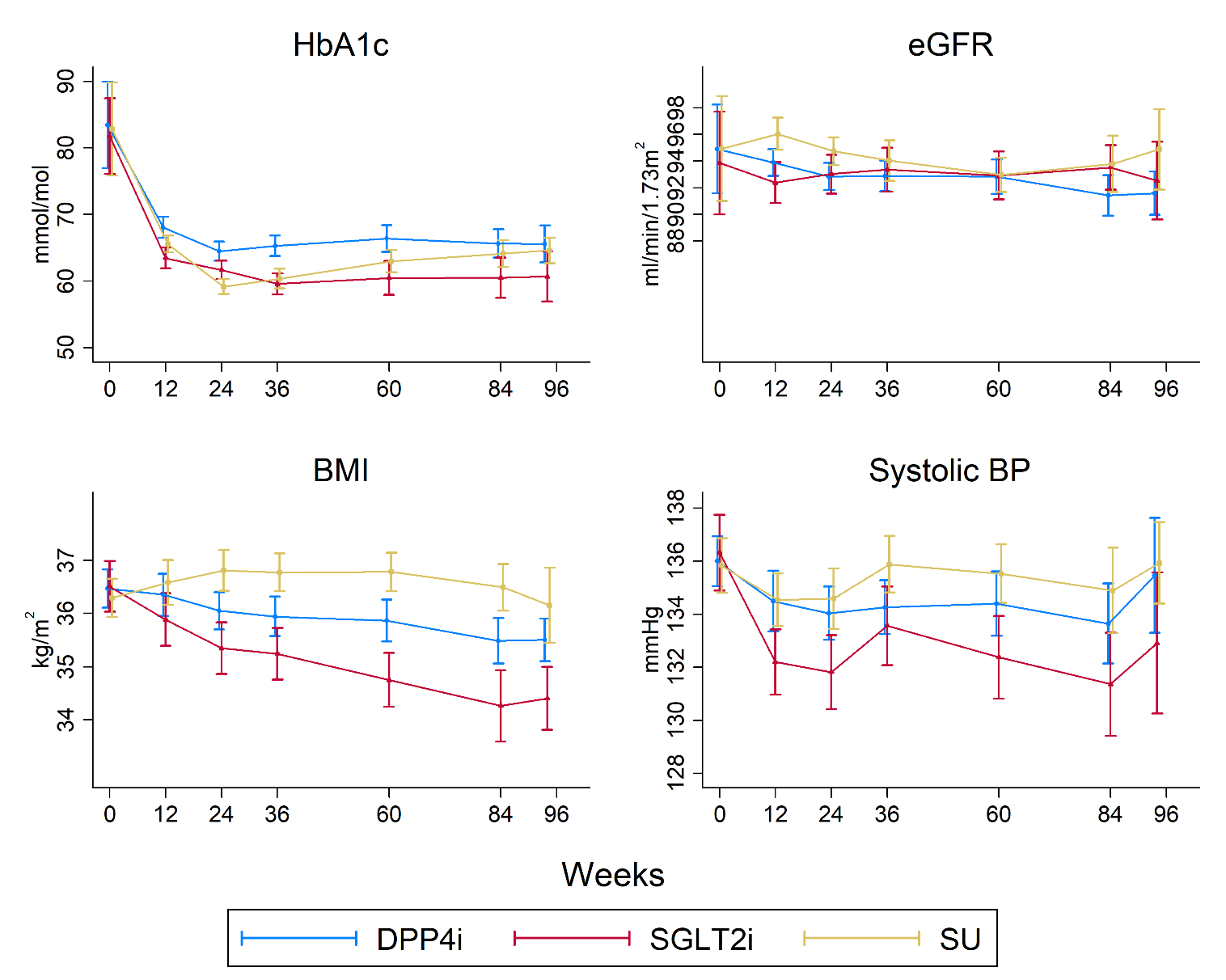
**

**Supplementary Figure 5: Mean (95% confidence intervals) of each clinical measure during treatment, for propensity score matched cohorts of individuals with missing data handled using multiple imputation, following intensification with DPP4i, SGLT2i and SU**

**
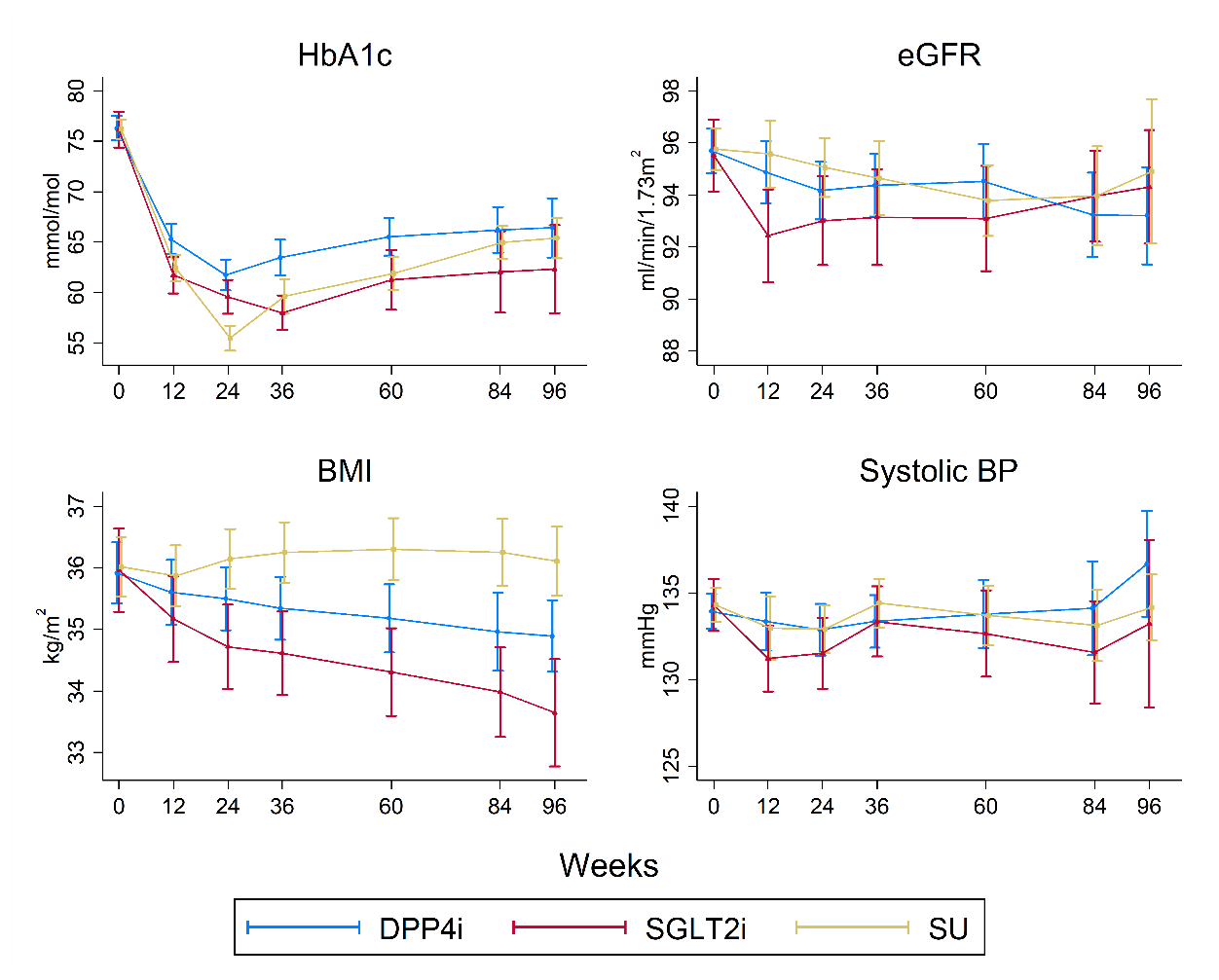
**

**Supplementary Figure 6: Mean (95% confidence intervals) of each clinical measure during treatment, for a propensity score matched cohort of individuals (using only individuals with baseline and follow-up measures for all of eGFR, HbA1c, BMI and BP), following intensification with DPP4i, SGLT2i and SU after metformin monotherapy**

**
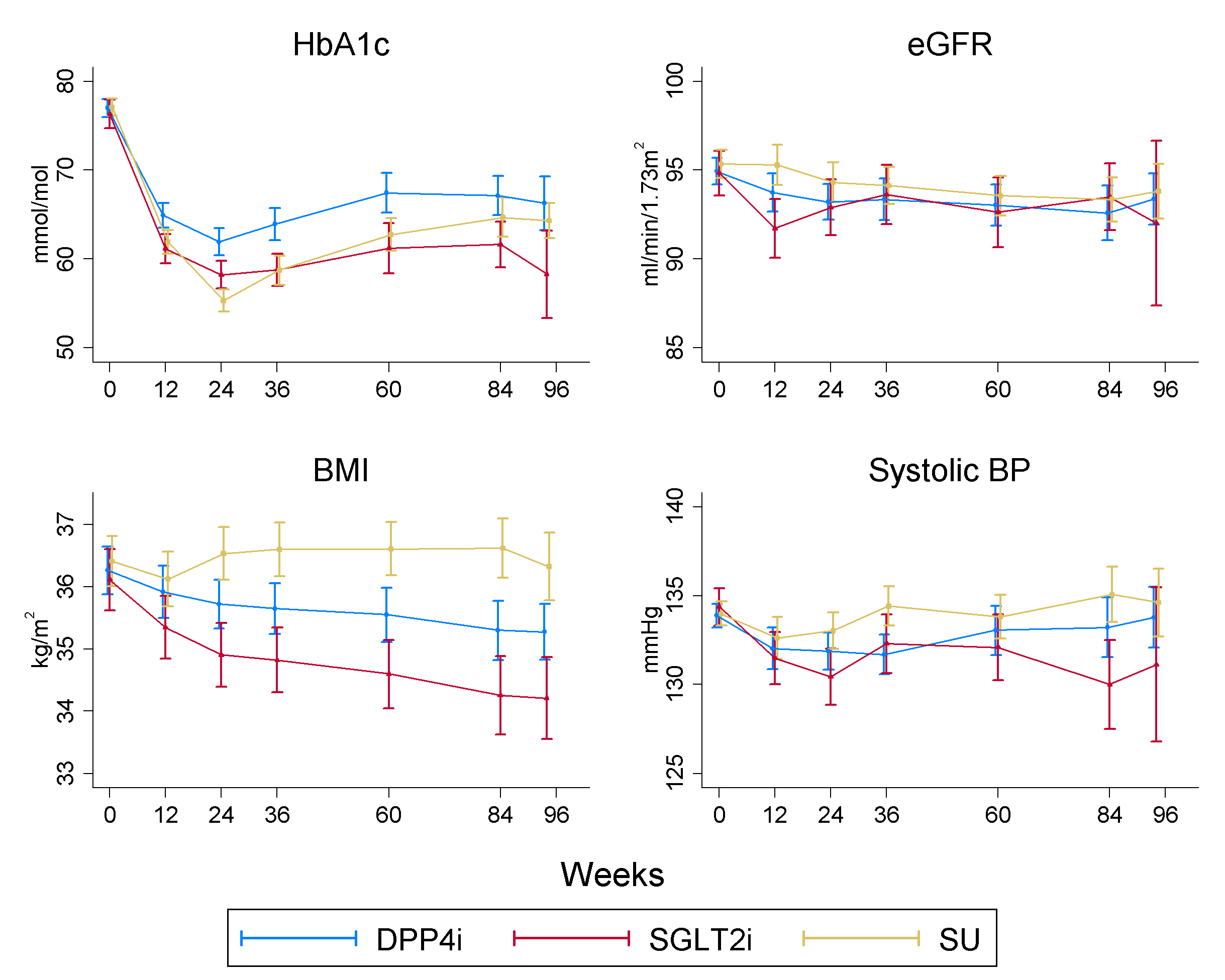

Supplementary Figure 7: Mean (95% confidence intervals) of each clinical measure during treatment, primary analysis repeated excluding individuals in the top 25^th^ percentile for the number of tests available during follow-up (eGFR, HbA1c, BMI and BP), following intensification with DPP4i, SGLT2i and SU after metformin monotherapy**

| **Measure** | **SU** | | **SGLT2i** | | **DPP4i** | |
| --- | --- | --- | --- | --- | --- | --- |
| **Week:** | **12** | **60** | **12** | **60** | **12** | **60** |
| **HbA1c *mmol/mol*** | 66.1  (64.8 - 67.3) | 64.2  (62.9 - 65.6) | 64.8  (63.1 - 66.4) | 61.6  (59.1 - 64.1) | 68.5  (67.1 - 69.9) | 67.1  (65.5 - 68.8) |
| **eGFR *ml/min/1.73m^2^*** | 95.5  (94.6 - 96.5) | 92.2  (91.0 - 93.5) | 91.8  (90.2 - 93.4) | 92.2  (90.5 - 93.9) | 93.5  (92.4 - 94.6) | 92.7  (91.6 - 93.7) |
| **BMI *kg/m^2^*** | 36.6  (36.2 - 37.1) | 36.7  (36.3 - 37.1) | 35.6  (35.2 - 36.1) | 34.7  (34.2 - 35.2) | 36.1  (35.7 - 36.4) | 35.6  (35.2 - 36.0) |
| **Systolic BP *mmHg*** | 133.8  (132.8 - 134.9) | 135.7  (134.7 - 136.7) | 132.6  (131.3 - 133.8) | 132.6  (131.2 - 134.1) | 133.6  (132.6 - 134.6) | 134.1  (133.0 - 135.2) |

**Supplementary table 12: Mean HbA1c, eGFR, BMI and systolic BP and 95% CI at 12 and 60 weeks for SU, SGLT2i and DPP4i treatment cohorts, intention to treat analysis**

|  | **EMPA-REG (2)** | **CANVAS (3)** | **DECLARE-TIMI (4)** | **CREDENCE (5)** | **Current study**^**^ |
| --- | --- | --- | --- | --- | --- |
| Mean age (years) | 63 | 63 | 64 | 63 | 56 |
| Female (%) | 28 | 36 | 37 | 34 | 40 |
| Mean HbA1c (mmol/mol)**^#^** | 65 | 66 | 67 | 67 | 76 |
| Mean Systolic BP (mmHg) | 135 | 136 | 135 | 140 | 134 |
| Mean BMI (kg/m^2^) | 31 | 32 | 32 | 31 | 34 |
| Mean eGFR (mls/min/1.73m^2^) | 74 | 77 | 86 | 56 | 92 |
| Cardiovascular disease (%) | 98.9^c^ | 65.6 | 40.8 | 50.4 | 11 |
| Retinopathy (%) |  | 21 |  | 43 | 16 |
| Heart failure (%) | 10.1 | 14.4 | 10.0 | 14.8 | 2 |
| Concomitant medications |  |  |  |  |  |
| Prevalent metformin users | 74 | 77 | 82 | 58**^a^** | 100 |
| ACE Inhibitor users | 80 | 80**^†^** | 81**^$^** | 100**^†^** | 54**^$^** |
| Statin users | 76 | 75 | 75^b^ | 69 | 73 |

**Supplementary Table 13: Comparison of individuals included in our HbA1c matched analysis to individuals included in main SGLT2 inhibitor cardiovascular outcome trials.**

Where studies have not provided overall means or percentage, we have provided the placebo group statistics. **^#^**converted from % if data not given, **^**^**For HbA1c matched cohort, **^a^** biguanides, **^†^**Renin-angiotensin-aldosterone system inhibitors, **^$^**ACE Inhibitors or Angiotensin receptor blockers, **^b^** Statin or ezetimibe, **^c^** Cardiovascular risk factors (includes coronary artery disease, history of myocardial infarction, coronary artery bypass graft, history of stroke, peripheral artery disease, coronary artery disease and cardiac failure )

Abbreviations: SU: Sulfonylurea, DPP4i: dipeptidyl peptidase 4 inhibitors, SGLT2i: Sodium-glucose co-transporter-2 inhibitors, HbA1c: Haemoglobin A1c, eGFR: estimated glomerular filtration rate, BMI: Body mass index, BP: Blood pressure, ACEI: Angiotensin converting enzyme inhibitor, ARB: Angiotensin 2 receptor blockers.

|  | **Category** | **SU** | **SU Original cohort** | **SGLT2i** | **SGLT2i original cohort** | **DPP4i** | **DPP4i original cohort** |
| --- | --- | --- | --- | --- | --- | --- | --- |
| **Counts** |  | 2,521 | **5,010** | 704 | **1,187** | 2,339 | **4,434** |
| **Age at baseline** | Years | 60.2 (12.6) | **61 (13)** | 55 (10.3) | **55 (10)** | 60.4 (12.3) | **61 (12)** |
| **BMI baseline** | kg/m^2^ | 31.9 (6.2) | **32 (6)** | 36.9 (7.0) | **37 (7)** | 33.2 (6.4) | **33 (7)** |
| **eGFR baseline** | ml/min/1.73m^2^ | 89.6 (10.3) | **89 (18)** | 96.4 (7.0) | **96 (13)** | 88.8 (9.8) | **88 (18)** |
| **Systolic BP at baseline** | mmHg | 132.9 (12.0) | **133 (14)** | 134.2 (12.1) | **134 (14)** | 133.7 (11.8) | **133 (14)** |
| **HbA1c at baseline** | mmol/mol | 80.2 (12.0) | **80 (21)** | 77.1 (10.2) | **77 (17)** | 73 (9.0) | **73 (16)** |
| **Time taking metformin prior to intensification** | Month | 39.9 (36.3) | **40 (37)** | 35.9 (33.7) | **36 (33)** | 43.9 (36.7) | **44 (37)** |
| **Gender** | Female, n (%) | 993 (39) | **1988 (39.7)** | 271 (38) | **474 (39.9)** | 904 (39) | **1745 (39.4)** |
| **Diagnosis of CVD** | n (%) | 351 (14) | **707 (14.1)** | 74 (11) | **119 (10)** | 298 (13) | **601 (13.6)** |
| **Diagnosis of HF** | n (%) | 89 (4) | **194 (3.9)** | 9 (1) | **24 (2)** | 68 (3) | **146 (3.3)** |
| **Diagnosis of retinopathy** | n (%) | 411 (16.0) | **868 (17.3)** | 101 (14) | **181 (15.2)** | 442 (19) | **861 (19.4)** |
| **Prescription for ARB or ACE inhibitor** | n (%) | 1361 (54.0) | **2711 (54.1)** | 386 (55) | **670 (56.4)** | 1301 (56) | **2490 (56.2)** |
| **Prescription for statin** | n (%) | 1752 (69.0) | **3530 (70.5)** | 483 (69) | **819 (69)** | 1768 (76) | **3387 (76.4)** |
| **Patient-level index of multiple deprivation** | 1 LEAST deprived | 208 (8) | **467 (9.3)** | 44 (6) | **93 (7.8)** | 163 (7) | **398 (9.0)** |
|  | 2 | 230 (9) | **485 (9.7)** | 50 (7) | **99 (8.3)** | 156 (7) | **378 (8.5)** |
|  | 3 | 267 (11) | **567 (11.3)** | 54 (8) | **117 (9.9)** | 197 (8) | **449 (10.1)** |
|  | 4 | 338 (13) | **643 (12.8)** | 57 (8) | **99 (8.3)** | 215 (9) | **427 (9.6)** |
|  | 5 MOST deprived | 280 (11) | **589 (11.8)** | 49 (7) | **81 (6.8)** | 224 (10) | **479 (10.8)** |
|  | Missing category | 1198 (48) | **2259 (45.1)** | 450 (64) | **698 (58.8)** | 1384 (59) | **2303 (51.9)** |
| **Smoking status** | Non-smoker | 923 (37) | **1883 (37.6)** | 274 (39) | **462 (38.9)** | 866 (37) | **1642 (37.0)** |
|  | Current | 426 (17) | **818 (16.3)** | 116 (16) | **193 (16.3)** | 394 (17) | **688 (15.5)** |
|  | Ex-smoker | 1165 (46) | **2297 (45.8)** | 314 (45) | **532 (44.8)** | 1078 (46) | **2102 (47.4)** |
|  | Missing category | 7 (0) | **12 (0.2)** | <5 | **N<5** | <5 | **N<5** |
| **Ethnicity** | White | 1040 (41) | **2052 (41.5)** | 297 (42) | **500 (42.1)** | 1053 (45) | **1944 (43.8)** |
| n (%) | South Asian | 114 (5) | **229 (4.6)** | 21 (3) | **31 (2.6)** | 73 (3) | **146 (3.3)** |
|  | Black | 63 (2) | **122 (2.4)** | <5 | **9 (0.8)** | 32 (1) | **61 (1.4)** |
|  | Other | 35 (1) | **59 (1.2)** | <5 | **5 (0.4)** | 10 (0) | **26 (0.6)** |
|  | Mixed | 9 (0) | **14 (0.3)** | <5 | **N<5** | 7 (0) | **16 (0.4)** |
|  | Missing category | 1260 (50) | **2534 (50.6)** | 380 (54) | **640 (53.9)** | 1164 (50) | **2241 (50.5)** |
| **Year started** | 2014 | 957 (38) | **2090 (41.7)** | 125 (18) | **217 (18.3)** | 670 (29) | **1390 (31.3)** |
| **follow-up** | 2015 | 828 (33) | **1668 (33.3)** | 195 (28) | **355 (29.9)** | 689 (29) | **1453 (32.8)** |
| n (%) | 2016 | 536 (21) | **989 (19.7)** | 245 (35) | **444 (37.4)** | 664 (28) | **1207 (27.2)** |
|  | 2017 | 200 (8) | **263 (5.2)** | 139 (20) | **171 (14.4)** | 316 (14) | **384 (8.7)** |

**Supplementary Table 14: Characteristics for people dropped from the eGFR sample due to missing baseline values and/or follow-up values. Compared to the study population at baseline for individuals intensifying treatment from metformin monotherapy with SU, SGLT2i or DPP4i between 2014-2017 (Table 1)**

|  | **Category** | **SU** | **SU Original cohort** | **SGLT2i** | **SGLT2i original cohort** | **DPP4i** | **DPP4i original cohort** |
| --- | --- | --- | --- | --- | --- | --- | --- |
| **Counts** |  | 2358 | **5,010** | 671 | **1,187** | 2210 | **4,434** |
| **Age at baseline** | Years | 60.5 (12.8) | **61 (13)** | 55.1 (10.5) | **55 (10)** | 60.6 (12.4) | **61 (12)** |
| **BMI baseline** | kg/m^2^ | 31.9 (6.2) | **32 (6)** | 37 (7.2) | **37 (7)** | 33.1 (6.3) | **33 (7)** |
| **eGFR baseline** | ml/min/1.73m^2^ | 89.1 (10.2) | **89 (18)** | 96.1 (6.9) | **96 (13)** | 88.1 (10.3) | **88 (18)** |
| **Systolic BP at baseline** | mmHg | 132.9 (12.2) | **133 (14)** | 134.3 (12.1) | **134 (14)** | 133.7 (11.8) | **133 (14)** |
| **HbA1c at baseline** | mmol/mol | 79.8 (10.3) | **80 (21)** | 76.6 (9.3) | **77 (17)** | 72.9 (8.5) | **73 (16)** |
| **Time taking metformin prior to intensification** | Month | 40.1 (36.7) | **40 (37)** | 35.6 (32.9) | **36 (33)** | 44.6 (37.4) | **44 (37)** |
| **Gender** | Female, n (%) | 932 (40) | **1988 (39.7)** | 259 (39) | **474 (39.9)** | 847 (38) | **1745 (39.4)** |
| **Diagnosis of CVD** | n (%) | 341 (14) | **707 (14.1)** | 74 (11) | **119 (10)** | 291 (13) | **601 (13.6)** |
| **Diagnosis of HF** | n (%) | 91 (4) | **194 (3.9)** | 12 (2) | **24 (2)** | 73 (3) | **146 (3.3)** |
| **Diagnosis of retinopathy** | n (%) | 382 (16) | **868 (17.3)** | 101 (15) | **181 (15.2)** | 412 (19) | **861 (19.4)** |
| **Prescription for ARB or ACE inhibitor** | n (%) | 1306 (55) | **2711 (54.1)** | 369 (55) | **670 (56.4)** | 1235 (56) | **2490 (56.2)** |
| **Prescription for statin** | n (%) | 1652 (70) | **3530 (70.5)** | 458 (68) | **819 (69)** | 1677 (76) | **3387 (76.4)** |
| **Patient-level index of multiple** | 1 LEAST deprived | 174 (7) | **467 (9.3)** | 39 (6) | **93 (7.8)** | 141 (6) | **398 (9.0)** |
| **deprivation** | 2 | 219 (9) | **485 (9.7)** | 44 (7) | **99 (8.3)** | 139 (6) | **378 (8.5)** |
|  | 3 | 243 (10) | **567 (11.3)** | 52 (8) | **117 (9.9)** | 174 (8) | **449 (10.1)** |
|  | 4 | 316 (13) | **643 (12.8)** | 58 (9) | **99 (8.3)** | 198 (9) | **427 (9.6)** |
|  | 5 MOST deprived | 261 (11) | **589 (11.8)** | 43 (6) | **81 (6.8)** | 206 (9) | **479 (10.8)** |
|  | Missing category | 1145 (49) | **2259 (45.1)** | 435 (65) | **698 (58.8)** | 1352 (61) | **2303 (51.9)** |
| **Smoking status** | Non-smoker | 885 (38) | **1883 (37.6)** | 247 (37) | **462 (38.9)** | 820 (37) | **1642 (37.0)** |
|  | Current | 395 (17) | **818 (16.3)** | 115 (17) | **193 (16.3)** | 378 (17) | **688 (15.5)** |
|  | Ex-smoker | 1074 (46) | **2297 (45.8)** | 309 (46) | **532 (44.8)** | 1012 (46) | **2102 (47.4)** |
|  | Missing category | <5 | **12 (0.2)** | <5 | **N<5** | <5 | **N<5** |
| **Ethnicity** | White | 977 (41) | **2052 (41.5)** | 286 (43) | **500 (42.1)** | 983 (44) | **1944 (43.8)** |
| n (%) | South Asian | 120 (5) | **229 (4.6)** | 20 (3) | **31 (2.6)** | 66 (3) | **146 (3.3)** |
|  | Black | 62 (3) | **122 (2.4)** | <5 | **9 (0.8)** | 31 (1) | **61 (1.4)** |
|  | Other | 35 (1) | **59 (1.2)** | <5 | **5 (0.4)** | 10 (0) | **26 (0.6)** |
|  | Mixed | 9 (0) | **14 (0.3)** | <5 | **N<5** | 6 (0) | **16 (0.4)** |
|  | Missing category | 1155 (49) | **2534 (50.6)** | 358 (53) | **640 (53.9)** | 1114 (50) | **2241 (50.5)** |
| **Year starting** | 2014 | 897 (38) | **2090 (41.7)** | 122 (18) | **217 (18.3)** | 617 (28) | **1390 (31.3)** |
| **follow-up** | 2015 | 761 (32) | **1668 (33.3)** | 183 (27) | **355 (29.9)** | 658 (30) | **1453 (32.8)** |
| n (%) | 2016 | 501 (21) | **989 (19.7)** | 231 (34) | **444 (37.4)** | 609 (28) | **1207 (27.2)** |
|  | **2017** | 199 (8) | **263 (5.2)** | 135 (20) | **171 (14.4)** | 326 (15) | **384 (8.7)** |

**Supplementary Table 15: Characteristics for people dropped from the HbA1c sample due to missing baseline values and/or follow-up values. Compared to the study population at baseline for individuals intensifying treatment from metformin monotherapy with SU, SGLT2i or DPP4i between 2014-2017 (Table 1)**

|  | **Category** | **SU** | **SU Original cohort** | **SGLT2i** | **SGLT2i original cohort** | **DPP4i** | **DPP4i original cohort** |
| --- | --- | --- | --- | --- | --- | --- | --- |
| **Counts** |  | 1870 | **5,010** | 423 | **1,187** | 1751 | **4,434** |
| **Age at baseline** | Years | 61.2 (13.2) | **61 (13)** | 56.3 (10.1) | **55 (10)** | 61.6 (12.8) | **61 (12)** |
| **BMI baseline** | kg/m^2^ | 31.6 (5.6) | **32 (6)** | 36.4 (7.3) | **37 (7)** | 32.8 (6.0) | **33 (7)** |
| **eGFR baseline** | ml/min/1.73m^2^ | 88.4 (15.1) | **89 (18)** | 95.2 (10.2) | **96 (13)** | 87.6 (15.2) | **88 (18)** |
| **Systolic BP at baseline** | mmHg | 133.7 (11.8) | **133 (14)** | 134.5 (10.2) | **134 (14)** | 134 (11.5) | **133 (14)** |
| **HbA1c at baseline** | mmol/mol | 80.1 (16.6) | **80 (21)** | 76.3 (13.2) | **77 (17)** | 73.2 (13.5) | **73 (16)** |
| **Time taking metformin prior to intensification** | Month | 39.3 (36.4) | **40 (37)** | 37 (32.2) | **36 (33)** | 46.3 (38.4) | **44 (37)** |
| **Gender** | Female, n (%) | 746 (40) | **1988 (39.7)** | 160 (38) | **474 (39.9)** | 660 (38) | **1745 (39.4)** |
| **Diagnosis of CVD** | n (%) | 267 (14) | **707 (14.1)** | 46 (11) | **119 (10)** | 231 (13) | **601 (13.6)** |
| **Diagnosis of HF** | n (%) | 94 (5) | **194 (3.9)** | 8 (2) | **24 (2)** | 71 (4) | **146 (3.3)** |
| **Diagnosis of retinopathy** | n (%) | 289 (15) | **868 (17.3)** | 70 (17) | **181 (15.2)** | 342 (20) | **861 (19.4)** |
| **Prescription for ARB or ACE inhibitor** | n (%) | 1007 (54) | **2711 (54.1)** | 244 (58) | **670 (56.4)** | 983 (56) | **2490 (56.2)** |
| **Prescription for statin** | n (%) | 1275 (68) | **3530 (70.5)** | 292 (69) | **819 (69)** | 1293 (74) | **3387 (76.4)** |
| **Patient-level index of multiple** | 1 LEAST deprived | 179 (10) | **467 (9.3)** | 31 (7) | **93 (7.8)** | 156 (9) | **398 (9.0)** |
| **deprivation** | 2 | 215 (11) | **485 (9.7)** | 38 (9) | **99 (8.3)** | 159 (9) | **378 (8.5)** |
|  | 3 | 216 (12) | **567 (11.3)** | 43 (10) | **117 (9.9)** | 178 (10) | **449 (10.1)** |
|  | 4 | 252 (13) | **643 (12.8)** | 39 (9) | **99 (8.3)** | 178 (10) | **427 (9.6)** |
|  | 5 MOST deprived | 238 (13) | **589 (11.8)** | 33 (8) | **81 (6.8)** | 206 (12) | **479 (10.8)** |
|  | Missing category | 770 (41) | **2259 (45.1)** | 239 (57) | **698 (58.8)** | 874 (50) | **2303 (51.9)** |
| **Smoking status** | Non-smoker | 698 (37) | **1883 (37.6)** | 168 (40) | **462 (38.9)** | 639 (36) | **1642 (37.0)** |
|  | Current | 309 (17) | **818 (16.3)** | 76 (18) | **193 (16.3)** | 280 (16) | **688 (15.5)** |
|  | Ex-smoker | 855 (46) | **2297 (45.8)** | 179 (42) | **532 (44.8)** | 832 (48) | **2102 (47.4)** |
|  | Missing category | 8 (0) | **12 (0.2)** | <5 | **N<5** | <5 | **N<5** |
| **Ethnicity** | White | 714 (38) | **2052 (41.5)** | 179 (42) | **500 (42.1)** | 774 (44) | **1944 (43.8)** |
| n (%) | South Asian | 95 (5) | **229 (4.6)** | 12 (3) | **31 (2.6)** | 64 (4) | **146 (3.3)** |
|  | Black | 56 (3) | **122 (2.4)** | <5 | **9 (0.8)** | 32 (2) | **61 (1.4)** |
|  | Other | 31 (2) | **59 (1.2)** | <5 | **5 (0.4)** | 16 (1) | **26 (0.6)** |
|  | Mixed | <5 | **14 (0.3)** | <5 | **N<5** | 9 (1) | **16 (0.4)** |
|  | Missing category | 969 (52) | **2534 (50.6)** | 226 (53) | **640 (53.9)** | 856 (49) | **2241 (50.5)** |
| **Year starting** | 2014 | 668 (36) | **2090 (41.7)** | 49 (12) | **217 (18.3)** | 400 (23) | **1390 (31.3)** |
| **follow-up** | 2015 | 563 (30) | **1668 (33.3)** | 92 (22) | **355 (29.9)** | 491 (28) | **1453 (32.8)** |
| n (%) | 2016 | 440 (24) | **989 (19.7)** | 152 (36) | **444 (37.4)** | 538 (31) | **1207 (27.2)** |
|  | 2017 | 199 (11) | **263 (5.2)** | 130 (31) | **171 (14.4)** | 322 (18) | **384 (8.7)** |

**Supplementary Table 16: Characteristics for people dropped from the BMI sample due to missing baseline values and/or follow-up values. Compared to the study population at baseline for individuals intensifying treatment from metformin monotherapy with SU, SGLT2i or DPP4i between 2014-2017 (Table 1)**

|  | **Category** | **SU** | **SU Original cohort** | **SGLT2i** | **SGLT2i original cohort** | **DPP4i** | **DPP4i original cohort** |
| --- | --- | --- | --- | --- | --- | --- | --- |
| **Counts** |  | 1128 | **5,010** | 363 | **1,187** | 1182 | **4,434** |
| **Age at baseline** | Years | 59.4 (13.0) | **61 (13)** | 55.1 (10.0) | **55 (10)** | 60.3 (12.6) | **61 (12)** |
| **BMI baseline** | kg/m^2^ | 31.8 (6.1) | **32 (6)** | 36.5 (7.7) | **37 (7)** | 33 (6.4) | **33 (7)** |
| **eGFR baseline** | ml/min/1.73m^2^ | 90 (14.5) | **89 (18)** | 96 (10.3) | **96 (13)** | 88.6 (14.8) | **88 (18)** |
| **Systolic BP at baseline** | mmHg | 132.2 (6.5) | **133 (14)** | 133.5 (5.4) | **134 (14)** | 132.3 (6.6) | **133 (14)** |
| **HbA1c at baseline** | mmol/mol | 80.9 (17.1) | **80 (21)** | 77.6 (13.5) | **77 (17)** | 73.9 (14.1) | **73 (16)** |
| **Time taking metformin prior to intensification** | Month | 39.1 (35.9) | **40 (37)** | 38.7 (34.7) | **36 (33)** | 45.5 (39.0) | **44 (37)** |
| **Gender** | Female, n (%) | 422 (37) | **1988 (39.7)** | 142 (39) | **474 (39.9)** | 473 (40) | **1745 (39.4)** |
| **Diagnosis of CVD** | n (%) | 146 (13) | **707 (14.1)** | 36 (10) | **119 (10)** | 147 (12) | **601 (13.6)** |
| **Diagnosis of HF** | n (%) | 46 (4) | **194 (3.9)** | <5 | **24 (2)** | 39 (3) | **146 (3.3)** |
| **Diagnosis of retinopathy** | n (%) | 159 (14) | **868 (17.3)** | 60 (17) | **181 (15.2)** | 213 (18) | **861 (19.4)** |
| **Prescription for ARB or ACE inhibitor** | n (%) | 549 (49) | **2711 (54.1)** | 191 (53) | **670 (56.4)** | 615 (52) | **2490 (56.2)** |
| **Prescription for statin** | n (%) | 759 (67) | **3530 (70.5)** | 251 (69) | **819 (69)** | 855 (72) | **3387 (76.4)** |
| **Patient-level index of multiple** | 1 LEAST deprived | 106 (9) | **467 (9.3)** | 25 (7) | **93 (7.8)** | 103 (9) | **398 (9.0)** |
| **deprivation** | 2 | 137 (12) | **485 (9.7)** | 39 (11) | **99 (8.3)** | 108 (9) | **378 (8.5)** |
|  | 3 | 127 (11) | **567 (11.3)** | 31 (9) | **117 (9.9)** | 132 (11) | **449 (10.1)** |
|  | 4 | 154 (14) | **643 (12.8)** | 37 (10) | **99 (8.3)** | 120 (10) | **427 (9.6)** |
|  | 5 MOST deprived | 133 (12) | **589 (11.8)** | 26 (7) | **81 (6.8)** | 147 (12) | **479 (10.8)** |
|  | Missing category | 471 (42) | **2259 (45.1)** | 205 (56) | **698 (58.8)** | 572 (48) | **2303 (51.9)** |
| **Smoking status** | Non-smoker | 408 (36) | **1883 (37.6)** | 136 (37) | **462 (38.9)** | 452 (38) | **1642 (37.0)** |
|  | Current | 214 (19) | **818 (16.3)** | 67 (18) | **193 (16.3)** | 193 (16) | **688 (15.5)** |
|  | Ex-smoker | 498 (44) | **2297 (45.8)** | 160 (44) | **532 (44.8)** | 536 (45) | **2102 (47.4)** |
|  | Missing category | 8 (1) | **12 (0.2)** | <5 | **N<5** | <5 | **N<5** |
| **Ethnicity** | White | 443 (39) | **2052 (41.5)** | 171 (47) | **500 (42.1)** | 531 (45) | **1944 (43.8)** |
| n (%) | South Asian | 53 (5) | **229 (4.6)** | 12 (3) | **31 (2.6)** | 33 (3) | **146 (3.3)** |
|  | Black | 32 (3) | **122 (2.4)** | <5 | **9 (0.8)** | 23 (2) | **61 (1.4)** |
|  | Other | 18 (2) | **59 (1.2)** | <5 | **5 (0.4)** | 12 (1) | **26 (0.6)** |
|  | Mixed | <5 | **14 (0.3)** | <5 | **N<5** | <5 | **16 (0.4)** |
|  | Missing category | 578 (51) | **2534 (50.6)** | 178 (49) | **640 (53.9)** | 578 (49) | **2241 (50.5)** |
| **Year starting** | 2014 | 386 (34) | **2090 (41.7)** | 43 (12) | **217 (18.3)** | 238 (20) | **1390 (31.3)** |
| **follow-up** | 2015 | 321 (28) | **1668 (33.3)** | 78 (21) | **355 (29.9)** | 318 (27) | **1453 (32.8)** |
| n (%) | 2016 | 267 (24) | **989 (19.7)** | 126 (35) | **444 (37.4)** | 355 (30) | **1207 (27.2)** |
|  | 2017 | 154 (14) | **263 (5.2)** | 116 (32) | **171 (14.4)** | 271 (23) | **384 (8.7)** |

**Supplementary Table 17: Characteristics for people dropped from the systolic blood pressure sample due to missing baseline values and/or follow-up values. Compared to the study population at baseline for individuals intensifying treatment from metformin monotherapy with SU, SGLT2i or DPP4i between 2014-2017 (Table 1)**
